# Cell-type-specific contributions to theta-gamma coupled rhythms in the hippocampus

**DOI:** 10.1162/netn_a_00427

**Published:** 2025-03-03

**Authors:** Spandan Sengupta, Afroditi Talidou, Jeremie Lefebvre, Frances K. Skinner

**Affiliations:** Krembil Brain Institute, Krembil Research Institute, University Health Network, Toronto, ON, Canada; Department of Biology, University of Ottawa, Ottawa, ON, Canada; Department of Mathematics, University of Toronto, Toronto, ON, Canada; Department of Physiology, University of Toronto, Toronto, ON, Canada

**Keywords:** Theta-gamma, Oscillations, Hippocampus, Rate model, Interneurons, Neural circuit model

## Abstract

Distinct inhibitory cell types participate in cognitively relevant nested brain rhythms, and particular changes in such rhythms are known to occur in disease states. Specifically, the coexpression of theta and gamma rhythms in the hippocampus is believed to represent a general coding scheme, but cellular-based generation mechanisms for these coupled rhythms are currently unclear. We develop a population rate model of the CA1 hippocampus that encompasses circuits of three inhibitory cell types (bistratified cells and parvalbumin [PV]-expressing and cholecystokinin [CCK]-expressing basket cells) and pyramidal cells to examine this. We constrain parameters and perform numerical and theoretical analyses. The theory, in combination with the numerical explorations, predicts circuit motifs and specific cell-type mechanisms that are essential for the coexistence of theta and gamma oscillations. We find that CCK-expressing basket cells initiate the coupled rhythms and regularize theta, and PV-expressing basket cells enhance both theta and gamma rhythms. Pyramidal and bistratified cells govern the generation of theta rhythms, and PV-expressing basket and pyramidal cells play dominant roles in controlling theta frequencies. Our circuit motifs for the theta-gamma coupled rhythm generation could be applicable to other brain regions.

## Author Summary

There are many different types of inhibitory cells in our brains that are differentially affected in disease. Concomitantly, coupled rhythms change in particular ways with disease. To help understand cell-type-specific changes in coupled rhythms, we develop a mathematical network model that is both respective of the cell type and also amenable to analyses. We focus on theta-gamma coupled rhythms in the hippocampus and include three different inhibitory cell types in our model circuits. By combining a theoretical analysis and numerical explorations, we find distinct contributions of these inhibitory cell types to coupled rhythms and predict motifs that are essential for the expression of theta-gamma coupled rhythms. Moving forward, we can leverage our model insights to help unravel cell-type contributions in disease states.

## INTRODUCTION

Oscillatory activities of wide-ranging frequencies are ubiquitous in many brain structures (Buzsáki, 2006). Theta oscillations, ranging from 3 to 12 Hz, are prominent local field potential (LFP) rhythms that are most robustly recorded from the CA1 region (Buzsáki, 2002). This rhythm plays an essential role in memory and spatial navigation (Buzsáki & Moser, 2013), with its frequency being related to the animal’s kinematics (Kropff, Carmichael, Moser, & Moser, 2021). High (7–12 Hz) and low (4–7 Hz) ranges of theta frequencies dependent on atropine sensitivity have long been observed (Buzsáki, 2002; Kramis, Vanderwolf, & Bland, 1975) and can be distinctly elicited by social or fearful stimuli, respectively (Tendler & Wagner, 2015). A single theta cycle has been considered to be “a functional unit capable of representing distinct temporal-spatial content at different phases” (M. A. Wilson, Varela, & Remondes, 2015), and higher frequency gamma (∼20–100 Hz) oscillations are nested within these theta cycles (e.g., see Scheffer-Teixeira et al., 2012). It has been suggested that such theta-gamma coupling is a general coding scheme with information processing implications (Colgin, 2015; Jensen & Colgin, 2007; Lisman, 2005). Dynamic modulation of theta-gamma coupled rhythms during sleep (Bandarabadi et al., 2019), visual exploration (Kragel et al., 2020), and association with working memory exists, and it is thus not surprising that there is increasing evidence of specific changes in theta-gamma coupling with memory impairments, disease, and its progression (Goutagny et al., 2013; Hamm, Héraud, Cassel, Mathis, & Goutagny, 2015; Karlsson, Lindenberger, & Sander, 2022; Kitchigina, 2018; Musaeus, Nielsen, Musaeus, & Høgh, 2020; Zhang et al., 2016). Yet, it remains unclear what cell circuit motifs and mechanism(s) are responsible for these coexpressed rhythms.

The plethora of various interneurons or inhibitory cell types with their particular biophysical characteristics and connectivities in the hippocampus make it challenging to figure out their particular contributions to functionally relevant theta and gamma activities (Buzsáki, 2006; Fishell & Kepecs, 2020; Freund & Buzsáki, 1996; Kepecs & Fishell, 2014; Klausberger & Somogyi, 2008; McBain & Fisahn, 2001; Pelkey et al., 2017). However, as theta-gamma coupled rhythms reflect cognitive processing, are potential disease biomarkers, *and* involve various interneuron types, we cannot ignore this inhibitory diversity. For example, in trying to understand the contributions of specific inhibitory cell types, it has long been noted that there is a separation between perisomatically and dendritically targeting interneuron types onto pyramidal cells (Freund & Buzsáki, 1996; McBain & Fisahn, 2001), leading to consideration of a dichotomy in inhibitory control of pyramidal cell excitability. Further, Freund (2003) has described another dichotomy of perisomatically targeting cholecystokinin (CCK)-expressing and parvalbumin (PV)-expressing basket cells in a “rhythm and mood” fashion (Freund, 2003; Freund & Katona, 2007)—PV-expressing basket cells contribute in a precise clockwork fashion, and CCK-expressing basket cells contribute in a highly modulatory way. Using and combining modern technologies, the distinctness of interneurons is being unraveled (Gouwens et al., 2020; Harris et al., 2018; Kessaris & Denaxa, 2023; Ratliff & Batista-Brito, 2020; Yao et al., 2021). Twenty-eight well-defined interneuron types have been identified, and bistratified cells, PV-expressing basket cells, and CCK-expressing basket cells are distinguishable. These collective studies help us bridge cell-type-focused experiments with theoretical and modeling endeavors.

Many mathematical models have been used to help determine mechanisms underlying theta, gamma, and theta-gamma coupled rhythms in the hippocampus (Ferguson & Skinner, 2015). For gamma rhythms, ING (interneuron network gamma), and PING (pyramidal ING) mechanisms have long been proposed (Whittington, Traub, Kopell, Ermentrout, & Buhl, 2000). For theta and theta-gamma coupled rhythms, network models have mostly focused on the dynamics between oriens-lacunosum-moleculare (OLM) interneurons, fast-spiking interneurons, and pyramidal cells (Ferguson & Skinner, 2015). Recently, building from tight experimental linkages, we showed that a large-enough network of pyramidal cells could initiate theta rhythms (Chatzikalymniou, Gumus, & Skinner, 2021). However, in general, high-dimensional spiking network model systems challenge our capacity to extract explicit mechanisms. To circumvent this, we here build and analyze a cell-type-specific mean field model that combines pyramidal cells and three distinct inhibitory cell types (bistratified cells and PV- and CCK-expressing basket cells) that were found to be essential for theta-gamma coupled rhythms from the cell type and cell-type interaction perspectives (Bezaire, Raikov, Burk, Vyas, & Soltesz, 2016; Chatzikalymniou, Sengupta, Lefebvre, & Skinner, 2022). This model leverages a population-scale description of the neural activity with experimentally constrained parameters to gather insight about theta-gamma coupling and theta frequency control in the hippocampus, while providing testable predictions.

We perform high-throughput simulations and obtain multiple constrained parameter sets for theta-gamma coupled rhythms indicating degeneracy. We systematically analyze the contribution of individual cell types involved in theta-gamma coupled oscillations by quantifying changes in spectral characteristics resulting from targeted stimulation of individual cell types, and we also examine whether particular cell types play dominant roles in the control of theta frequency. We perform a theoretical analysis, which, in combination with numerical explorations, predicts cell types and motifs essential for the coexistence of theta and gamma oscillations. We find that circuit motifs of pyramidal and bistratified cells play a governing role in the theta rhythms, with CCK-expressing and PV-expressing basket cells together with pyramidal cells contributing to gamma rhythms. Taken together, our results show that CCK-expressing basket cells act to initiate theta-gamma coupled rhythms by their ability to control pyramidal cell activity via disinhibition, and we present a general two-phase process by which theta-gamma coupled rhythms arise in network motifs.

## RESULTS

### Building and Exploring a Population Rate Model (PRM) of Theta-Gamma Coupled Rhythms

**Figure 1.**
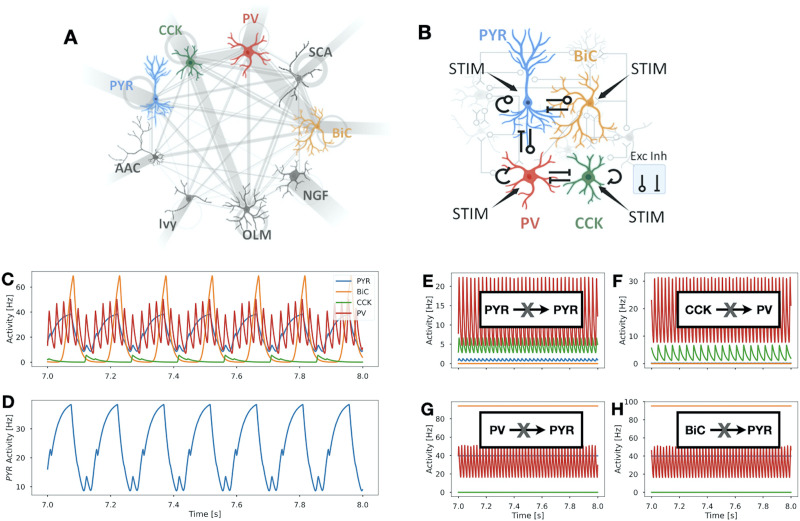
Schematics of microcircuit models of CA1 hippocampus and PRM output example. (A) Stylized schematic showing the nine different cell types that are present in a detailed circuit model of CA1 hippocampus (Bezaire et al., 2016). These cell types are pyramidal cells, paravalbumin-expressing basket cells, axo-axonic cells, bistratified cells, CCK-expressing basket cells, Schaeffer collateral-associated cells, OLM cells, neurogliaform cells, and Ivy cells. They are referred to as PYR, PV, AAC, BiC, CCK, SCA, OLM, NGF, Ivy, respectively. Connections between the different cell types are represented by gray lines between them. Gray circles by a given cell type indicate that they are interconnected, and incoming gray lines to the different cell types represent external drives. The thickness of the lines and circles is representative of the relative strength of these various connections. (B) Stylized schematic of the reduced model with four cell types (PYR, BiC, CCK, PV) and excitatory (Exc) and inhibitory (Inh) connections between them with possible inputs (STIM) to each cell type. The rationale used to consider this reduced model is given in the main text. (C) Activity of the four cell types (PYR, BiC, CCK, PV) in the PRM at baseline (i.e., when no STIM is applied to any of the cell types) over a 1-s interval. Colors match those shown in the schematic of (B) of the four cell-type circuit. The synaptic weight parameter values used in this simulation are from Set 4 in Supporting Information Table S1. (D) Activity of only PYR showing theta-gamma coupled rhythms. PYR output is analyzed as an LFP representation. (E–H) Removal of connections between cell types as schematized in each panel. These connections were previously shown to be required for theta-gamma coupled rhythms. See the text for further details.

In order to disambiguate the respective role of cell types and their mutual connectivity motifs in the generation and control of theta-gamma coupled rhythms in the CA1 hippocampus, we developed a reduced, system-level mean field PRM. This model combines connections and cell types that were identified as critical for the existence of theta-gamma rhythms based on previous work using more detailed circuit models with nine different cell types—pyramidal cells (**PYR**), bistratified cells (**BiC**), PV-expressing basket cells (**PV**), CCK-expressing basket cells (**CCK**), axo-axonic cells, Schaeffer collateral-associated cells, Ivy cells, neurogliaform cells, and OLM cells (Bezaire et al., 2016; Chatzikalymniou et al., 2021, 2022). Specifically, we previously showed that theta rhythms are lost with the removal of certain connections, and we rationalized the use of a four cell-type (PYR, BiC, CCK, PV) microcircuit (Bezaire et al., 2016; Chatzikalymniou et al., 2022) to examine theta-gamma coupled rhythms in the CA1 hippocampus. In Figure 1A–B, we show schematics of the cell types and interconnections used in previous detailed microcircuit models of the CA1 hippocampus (Figure 1A) and the four cell-type microcircuit used here (Figure 1B).

We built a PRM of the four-cell microcircuit depicted in Figure 1B and explored it using a combination of techniques. Mathematical equations and parameter descriptions are provided in the Model and Methods section. This PRM is novel relative to other mean field models in its incorporation of distinct cell types, particular interconnectivities, and dynamics that speak of both theta and gamma oscillations and their coexistence. Its deliberate simplicity, compared with the other detailed models, represents an important advantage as it enables a thorough exploration of parameter space with constrained values and a detailed mathematical characterization of different cell-type contributions. Since extracellular LFP recordings are typically done in the pyramidal cell layer, with PYR constituting the vast majority of the cells in the network, we consider PYR activity as representing the LFP output. The PRM exhibits LFP oscillations within gamma and theta frequency bands that are coupled with one another. An example is shown in Figure 1C. We show the activities of all four cell types in Figure 1C and only PYR in Figure 1D to more easily visualize the LFP representation of theta-gamma coupled rhythms.

The results are presented as follows. We first leverage a genetic algorithm to obtain constrained parameter sets. This reveals degeneracy in the expression of theta-gamma coupled rhythms and variability of the different connection types. That is, there are many different sets of synaptic weights that produce theta-gamma coupled rhythms. We then perform theoretical analyses on subsystems of the PRM. The use of the constrained parameter sets greatly reduces the dimensionality of the system and allows us to obtain cellular insights into theta and gamma rhythm generation. We then carry out extensive numerical simulations and are able to expose contributions by the different cell types. In particular, we show that CCK exhibits multiple roles that include its ability to initiate the expression of theta-gamma coupled rhythms. In the last section, we bring all our results together and present essential cell-type-specific motifs underlying the expression of theta-gamma coupled rhythms.

### Using a Genetic Algorithm Exposes Degeneracy in Theta-Gamma Coupled Rhythm Expression

The coexistence of theta and gamma oscillations, as illustrated in Figure 1D, results from a combination of excitation-inhibition motifs and delayed feedback. The frequency and power of these theta-gamma coupled rhythms are controlled by a combination of parameters. To determine which parameter combinations allow theta-gamma coupled rhythms in the PRM, we first applied constraints from the experimental literature for each of the four different cell types, while setting parameters to values that produce coupled oscillations within rationalized constraints (see the Model and Methods section). In doing this, we ensured that the overall dynamics of the network and relative differences in cell type properties were both taken into consideration.

One such important constraint is the manifest loss of the theta activity following connection removal. Specifically, theta oscillations should no longer be present whenever connections between PYR and PYR, from CCK to PV, from PV to PYR, and from BiC to PYR are removed (Chatzikalymniou et al., 2022). This constraint guided our parameter search: From an initial set of parameter values, we used a genetic algorithm search in the parameter space of the synaptic weights (*w*) and obtained 200 sets of synaptic weights satisfying the above criterion. For the example parameter set of Figure 1C, we show in Figure 1E–H that the constraint of connection removals is satisfied.

**Figure 2.**
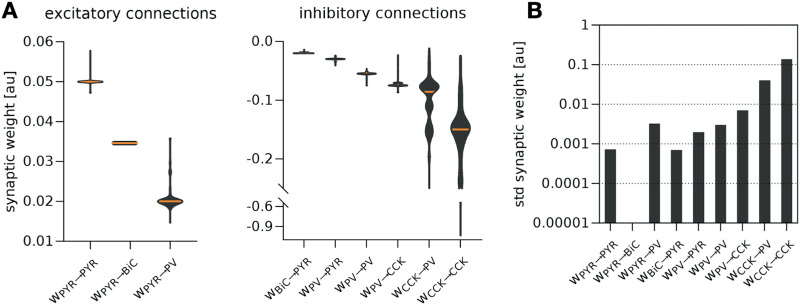
Distributions of constrained synaptic weights. (A) Distribution of synaptic weights (*w*) for each of the nine different connections in the PRM separated for excitatory (left) and inhibitory (right), from the 200 constrained parameter sets of *w* found using a genetic algorithm. The orange line depicts the median, and distributions are depicted as violin plots. (B) Bar graph showing the stds of *w* for each of the nine different connections.

The multiple sets of *w* obtained illustrate the degeneracy of the model system producing theta-gamma coupled rhythms. The distribution of these 200 sets of *w* is shown in Figure 2A. The PYR → PYR connection had the largest synaptic weight for excitatory connections, and for inhibitory connections, the largest synaptic weights involved CCK: CCK → CCK and CCK → PV. The variability differs for the nine different connection types, and this can be appreciated by observing their standard deviations (stds) shown in the bar graph histogram of Figure 2B. As the rationale involving the maximal firing of CCK was subjective (see PRM Hypotheses and Experimental Constraints in the Supporting Information), and noting the large std for the synaptic weights involving CCK, we reran the genetic algorithm with an adjusted constraint regarding CCK. The outcome is shown in Supporting Information Figure S2. The relative differences of stds remained, but the CCK-related connections were more strongly affected. Overall, these results start to suggest that CCK may have a more specific role relative to the other cell types.

From the 200 parameter sets shown in Figure 2A, we applied clustering techniques and selected 10 parameter sets (see the Model and Methods section for details) for further analyses. These 10 chosen parameter sets (*Sets 0–9*) include a variety of synaptic weight parameter values that generate theta-gamma coupled rhythms in the PRM, and are representative of the degeneracy (see the Model and Methods subsection Genetic Algorithm). Values of the synaptic weights for each of the nine different connections are given in Supporting Information Table S1. With these 10 constrained parameter sets in hand, we are able to greatly reduce the dimensionality of our theoretical analyses and to do extensive and systematic numerical explorations.

### Theoretical Analyses Predict Circuit Motifs Underlying Theta and Gamma Rhythms

**Figure 3.**
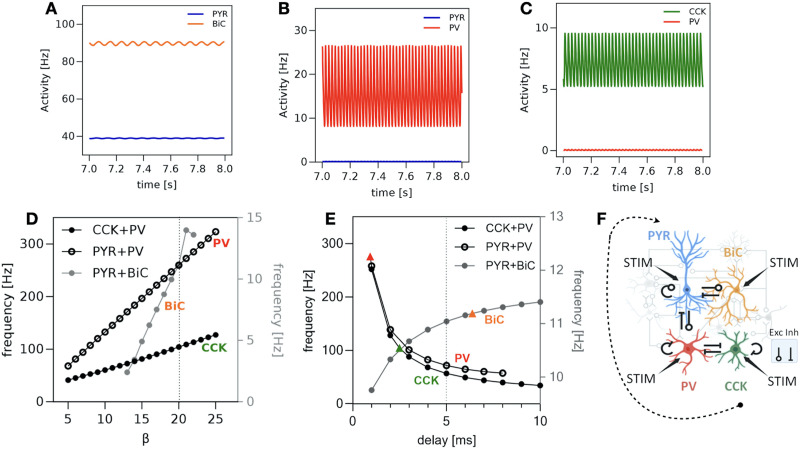
Theoretical analyses of PRM subsystems. (A) Activity of the two cell types (PYR, BiC) in PYR + BiC subsystem over a 1-s interval showing theta rhythms. (B) Activity of the two cell types (PYR, PV) in the PYR + PV subsystem. PYR is silent while PV exhibits gamma frequencies. (C) Activity of CCK and PV in the CCK + PV subsystem. CCK oscillates with gamma frequencies while PV demonstrates only a small amplitude and activity. (D) Frequencies of BiC, CCK, and PV are plotted for different values of *β*. (E) Frequencies plotted for BiC, PV, and CCK as a function of different delay values. The colored triangles show the bifurcation points (i.e., delays) for the three subsystems (PYR + BiC, PYR + PV, and CCK + PV). In panels (D) and (E), the frequencies are plotted for the cell type with the highest activity (between the two cell types of the subsystem). (F) Schematic illustration of the inhibition mechanism of PYR initiated by CCK. *Set 0* parameter values were used in the calculations for these plots. In panels (A), (B), and (C), *β* = 20 and *τ* = 5 ms.

To gain insights into the cell types and connectivity motifs contributing to the generation and interaction of theta and gamma rhythms, we leveraged a linear stability analysis. We did this by systematically examining connectivity motifs responsible for generating oscillations in both the theta and gamma frequency bands. To achieve this, we dissected the PRM (see Equations 1a–1d in the Model and Methods section) into three distinct and independent motifs—specifically PYR and BiC (PYR + BiC), PYR and PV (PYR + PV), and CCK and PV (CCK + PV)—each corresponding to a subsystem of dimension two. These three motifs are independent in the sense that, even if they may share the same cell types, they do not interact with each other. This approach enabled us to examine the behavior of each motif, thereby enhancing our understanding of their contributions to the theta and/or gamma activity. Additional details can be found in the Model and Methods section. Activities of the cell types for the three subsystems as a function of time are shown in Figure 3A–C. The dynamics of PYR + BiC generates rhythms within the theta band, whereas both PYR + PV and CCK + PV motifs independently generate gamma frequency rhythms.

While many parameters could have been considered, we focused our analysis on the roles of the response gain *β* and the synaptic and axonal time delay *τ*, given their well-known implication in promoting neural synchrony (Dhamala, Jirsa, & Ding, 2004; Erneux, 2009). We found that varying the value of *β* changes the frequencies of the different cell types. Specifically, frequency increases with increasing *β* (Figure 3D). This relation comes directly from the stability analysis (see Equations 16a–16b in the Model and Methods section). The time delay was found to influence the oscillatory behavior of each subsystem. To study its role in the generation of the theta and gamma activity, we fix *β* = 20 and further analyze the frequencies of each of the three subsystems. In Figure 3E, the frequencies of the three subsystems are depicted as a function of delays. For each subsystem, we plotted the activity of only one cell type because either the second cell type remains silent or exhibits a very small amplitude (see Figure 3A–C). Notably, the frequency of PYR + BiC consistently remains in the theta band across all selected delay values. In contrast, CCK + PV and PYR + PV subsystems demonstrate higher frequencies than gamma for small delays. As delays increase, the associated frequency values decrease, ultimately settling within the gamma band. The linear stability analysis further reveals that these oscillations emerge through Hopf bifurcations. We interpret these results as a demonstration that the independent subsystems examined (i.e., PYR + PV, PV+CCK, PYR + BiC) are able to generate theta or gamma rhythms on their own and then coupled rhythms arise once these subsystems are combined. The fact that only one cell type exhibits oscillations in the different panels of Figure 3A–C indicates that (a) input from the second, nonoscillating cell type is necessary for the rhythms to emerge in each of these subsystems and that (b) resulting oscillations emerge due to the recurrent interactions among the oscillating cell types. To maintain simplicity in our model, for the remainder of the study, we fix the delay at a value at which our analysis predicts the coexistence of both theta and gamma frequencies. We choose 5 ms as this is reasonable considering physiological constraints (see the Model and Methods section).

In Figure 3B, we observe a high activity of PV in the PYR + PV subsystem within the gamma band, while PYR remains silent. Similarly, in the CCK + PV subsystem (Figure 3C), PV is almost silent, while CCK is active at a frequency also in the gamma band. This suggests a potential mechanism where CCK is implicated in the initiation of gamma frequencies inhibiting PV, and PV subsequently inhibits PYR. This mechanism is schematized in Figure 3F. The results depicted in Figure 3 are from *Set 0*, but hold for all of the 10 parameter sets (see parameter values in Supporting Information Table S1).

### Numerical Explorations Support Theoretical Predictions for Circuit Motifs Underlying Theta and Gamma Rhythm Generation

To assess the susceptibility of each cell type to the expression of theta-gamma coupled rhythms, we systematically explored our four-cell circuit by applying a stimulus (i.e., STIM; see Figure 1B) to each of the four different cell types, using the 10 chosen parameter sets (Supporting Information Table S1). We performed simulations for a wide range of STIM values and measured stimulation ranges over which theta-gamma coupled rhythms persist to assess the robustness of the coupling and the roles played by the different cell types in influencing their existence.

**Figure 4.**
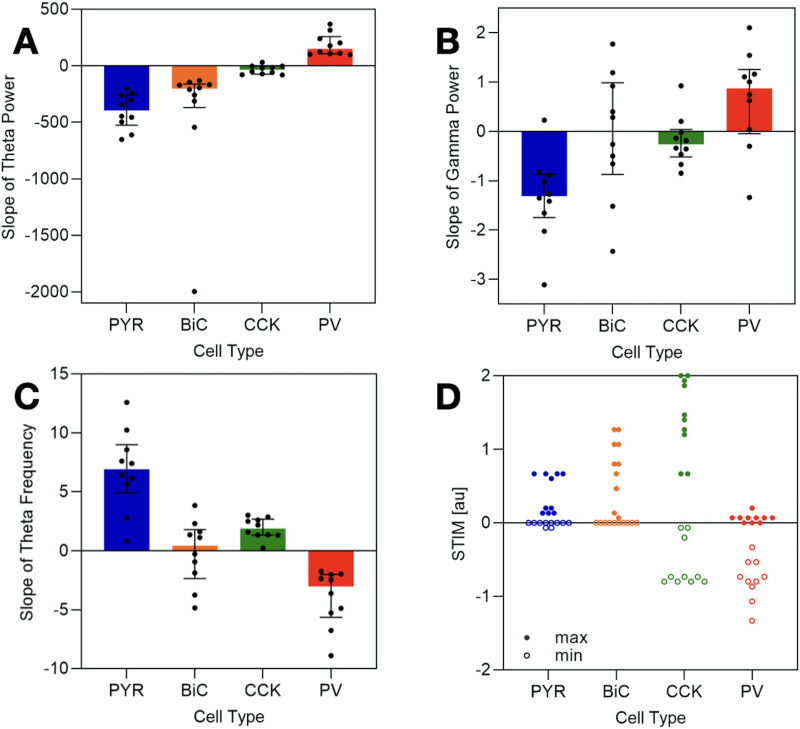
Cell type sensitivities. STIM values are applied to each cell type for each of the 10 parameter sets (Supporting Information Table S1). Theta and gamma powers and theta frequency are extracted when theta-gamma coupled rhythms are present. Plots show the distribution of the slope of the (A) theta power, (B) gamma power, and (C) theta frequency, with increasing STIM to each cell type. The slope is derived from a linear best fit (see Supporting Information Figure S3) through the points where the system exhibits theta-gamma coupled rhythms (see the Model and Methods section for further details). For all of the plots in (A–C), the bars extend from the first quartile to the third quartile of the data, shown with the median. The colors are representative of the particular cell type (see Figure 1B). (D) Minimum (min) and maximum (max) STIM values to the different cell types that allowed theta-gamma coupled rhythms to be expressed. Statistical significances are provided in Supporting Information Tables S4 and S5.

The stability analysis predicted that PYR + BiC circuits can function as theta frequency rhythm generators, but not any of the other two cell subsystems. For gamma rhythms, either PYR + PV or CCK + PV (or both) leads to gamma frequency rhythm expression. To consider this in the full four-cell circuit, we examined STIM values that preserved the theta-gamma regime and determined how sensitive the spectral properties of the coexisting oscillations were to selective stimulation. We considered theta-gamma coupled rhythms to be preserved (i.e., sufficiently present) if both theta and gamma rhythms had powers that were at least 25% of a reference when STIM was zero (baseline). We quantified theta and gamma powers from the theta-gamma coupled rhythms and calculated the slope of a linear fit to theta or gamma power plots with STIM. This is illustrated in Supporting Information Figure S3. In Figure 4, we plot the values of these fitted slopes for each cell type for the theta power (Figure 4A) and gamma power (Figure 4B).

We note that theta power increases (i.e., positive values in Figure 4A) existed only with increasing STIM to PV. PYR and BiC both showed theta power decreases, and there was minimal effect with CCK. Considering their medians, maximal absolute changes occurred for PYR and BiC, although BiC showed a large variability. This is interesting since our theoretical analyses above predicted that only PYR + BiC circuits, and not the other two-cell circuit subsystems, produce theta frequency rhythms (see Figure 3E). Given that our numerical explorations showed that the theta power changed maximally for PYR and BiC when they were stimulated indicates that the magnitude of the theta power change with stimulation in our numerical explorations of the four-cell system is consistent with the expression of theta rhythms in our theoretical analyses of the subsystems. For gamma power, the maximal absolute changes occurred for PYR and PV, as shown in Figure 4B. Given the consistency between the numerics and the stability analysis outcome for theta rhythms, in a similar vein, our numerical exploration observations suggest that the PYR + PV subsystem could be more key for gamma rhythm generation relative to the CCK + PV subsystem. However, as the gamma powers are much smaller than the theta powers (see Supporting Information Figures S3 and S4), more investigation is needed to extract further particular contributions of cell types for the fast rhythms.

Given the functional importance of theta frequencies, we examined how they changed, as part of the theta-gamma coupled rhythms, when the different cell types were stimulated. In Figure 4C, we show a quantification of how much theta frequency changed and note that the largest absolute changes occurred with PYR and PV stimulation. BiC showed both increases and decreases in frequency with stimulation.

### CCK Plays Multiple Roles in Theta-Gamma Coupled Rhythm Expression

We measured the range of STIM values associated with sufficient theta-gamma coupling. In Figure 4D, we present the minimum and maximum STIM values for each cell type. It is evident that CCK exhibits the widest range, indicating that theta-gamma coupled rhythms can be present for a wider range of external inputs compared with the other cell types in the network. PYR has the smallest range (see Supporting Information Table S4 for statistical significance).

**Figure 5.**
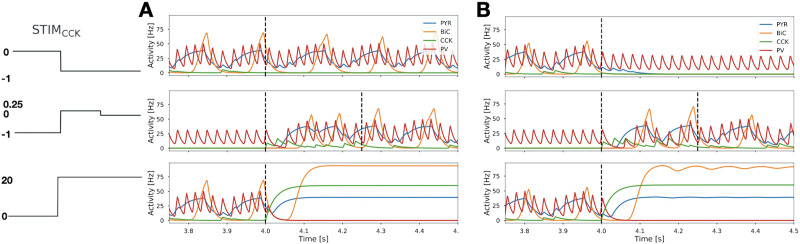
Effects of CCK perturbation on theta-gamma coupled rhythms. (*Top plots*) CCK is removed from the circuit at 4.0 s by changing STIM_*CCK*_ to −1 (vertical dashed line), leading to the continuation of the theta-gamma coupled rhythms for *Set 4* (A), or to loss of coupled rhythms for *Set 5* (B). (*Middle plots*) CCK is removed from the circuit at the start of the simulation, and at 4.0 s, CCK is “added back” to the circuit by changing STIM_*CCK*_ to 0.25 for a brief interval (as delineated by the first vertical dashed lines) and then returned to the baseline (i.e., STIM_*CCK*_ to 0) at the second vertical dashed line. Theta-gamma coupled rhythms are expressed when CCK is added back to the circuit. (*Bottom plots*) The CCK activity is strongly enhanced in the circuit at 4.0 s by changing STIM_*CCK*_ to 20 (vertical dashed line), leading to the loss of the theta-gamma coupled rhythms for *Set 4* (A) or *Set 5* (B). Representations of the changes in STIM_*CCK*_ are shown on the left-hand side for each row of plots.

Given the larger synaptic weights and variability involving CCK, as exposed by use of a genetic algorithm, we thought that CCK may have more specific roles compared with other cell types. We thus carried out additional investigations that revealed multiple CCK contributions. We removed CCK by silencing them using a strong inhibitory STIM, either at the beginning (*middle plots* of Figure 5) of the simulation or during (*top plots* of Figure 5) ongoing theta-gamma coupled rhythms in the 10 sets. Removal at the start of the simulation (*middle plots*) consistently prevented theta-gamma coupling—note that all cell types begin in a quiescent state across all simulations (see the Model and Methods section). Introducing a CCK “burst” to the circuit, previously devoid of theta-gamma coupled rhythms due to the absence of CCK, resulted in the expression of theta-gamma coupling (*middle plots*). Upon examining the timing of CCK and PV following a CCK “burst,” we found that CCK firing precedes PV firing for all 10 sets (not shown). These results support the notion that CCK is central in initiating the expression of theta-gamma coupled rhythms, as suggested from our theoretical analyses (see Figure 3F). However, once coupled rhythms are expressed, CCK activity is not necessarily required. In *Sets 3*, *5* (shown in Figure 5B), and *8*, where CCK activity was needed for the continuation of theta-gamma coupled rhythms (*top plot*), we observe larger connections between PYR and PV (see Supporting Information Table S1), suggesting that a sufficiently active PYR can sustain theta-gamma coupled rhythms without CCK activity in the circuit.

We further examined whether there would be any change in the regularity of the ongoing theta rhythm with or without CCK during theta-gamma coupled rhythms. Analyzing intervals between PYR peaks (i.e., the cycle period of the LFP representation), we observed a notable increase in the std of these intervals when CCK was absent from the circuit producing theta-gamma coupled rhythms in the majority of sets (see Supporting Information Table S3 and Supporting Information Figure S5). This suggests that CCK could play a role in regularizing the spectral features of theta activity.

Lastly, strong activation of CCK silences PV, preventing coupled rhythms as gamma rhythms are no longer present in PV and they cannot be propagated to PYR from CCK (see *bottom plots* of Figure 5). Thus, the level of CCK activity, along with the activity of the other cells in the circuit, can serve as a determinant for the expression of theta-gamma coupled rhythms, suggesting a potential “switching” role for CCK.

### Bringing It All Together

Due to the combined insights from our theoretical analyses and numerical explorations of the PRM, we can assign particular roles to the different cell types.

**Figure 6.**
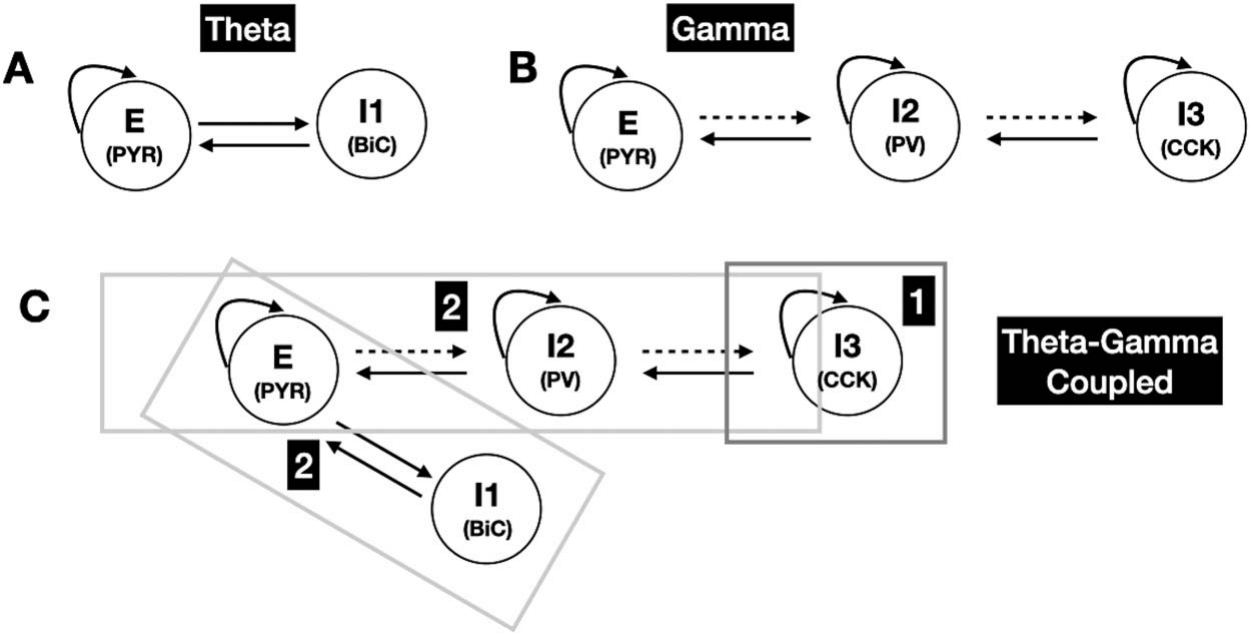
Predicted motifs for coupled rhythms. As derived from theoretical and numerical analyses of the four-cell circuit shown in Figure 1B with ***E*** = PYR, ***I1*** = BiC, ***I2*** = PV, ***I3*** = CCK. (A) The motif for the theta rhythm generation, (B) the motif for the gamma rhythm generation, and (C) the four-cell motif for the theta-gamma coupled rhythm generation. It occurs in two phases as shown in (C). In ***Phase 1***, ***I3*** **(*CCK*)** initiates the coupled rhythm by being able to disinhibit ***E*** **(*PYR*)**, which then allows theta rhythms to be generated (via ***E*** **[PYR]** and ***I1*** **[BiC]**) together with gamma rhythms (via ***E*** **[PYR]**, ***I1*** **[BiC]**, and ***I2*** **[PV]**) in ***Phase 2***. The dashed connection lines mean that they are not critical for the system to be able to express theta-gamma coupled rhythms—see Supporting Information Table S2. These motifs imply that at least three different inhibitory cell types (I1, I2, I3) need to be part of the circuitry for coupled rhythms to be expressed from quiescent states.

PYR and BiC play the role of theta rhythm generators. This is schematized in Figure 6A. This is based on the theoretical analyses of the subsystems in which only the PYR + BiC subsystem led to rhythms in the theta frequency band as fully supported by the numerical simulations of the full four-cell system that showed that PYR and BiC more strongly affected the theta power relative to the other cell types. CCK contributes to the gamma rhythm portion of theta-gamma coupled rhythms, serving as “initiators” of the gamma rhythm (see Figure 5), with PV contributing to the gamma by “applying” it to PYR. This is schematized in Figure 6B. Gamma rhythms can emerge from PYR + PV or CCK + PV subsystems. PV acts as an “enhancer” of both theta and gamma. That is, it is the only cell type that leads to an increase in *both* theta and gamma power with increasing stimulation (see Figures 4A and 4B).

Given the described roles for the different cell types, we expect that when the PV → PYR connection is removed, there would be no gamma (as it is coming from PV and CCK via PV) and, thus, no theta-gamma coupled rhythms. However, with the removal of the PYR → PV connection type, we expect that theta-gamma coupled rhythms might still be possible as theta could still exist due to the PYR + BiC subsystem, and gamma could still “circulate,” as schematized in Figure 3F. In Supporting Information Table S2, we see that theta-gamma coupled rhythms can be present for some of the parameter sets. Theta-gamma coupled rhythms can remain when the PV → CCK connection is removed (see Supporting Information Table S2), which makes sense since CCK can initiate the coupled rhythms. If PYR, PV, and BiC cells are quiescent, or not active enough, CCK is needed to initiate theta-gamma coupled rhythms (see *middle plots* of Figure 5). However, it is possible that CCK is not needed to maintain theta-gamma coupled rhythms if the different cells’ histories are such that they are active enough (see the *top plot* of Figure 5A). However, CCK clearly contributes to the regularity of the theta rhythm (see Supporting Information Table S3).

We thus present a two-phase process for the generation of theta-gamma coupled rhythms in the hippocampus, as schematized in Figure 6C. In the first phase, CCK generates gamma rhythms to initiate the theta-gamma coupled rhythm process. It can do this prior to PV contributing to gamma rhythms in light of its intrinsic properties (higher input resistance) and its surrounding milieu (see Supporting Information Figure S1). Supporting Information Figure S6 shows examples from two of the parameter sets illustrating CCK peaking before PV. In the second phase, theta is generated (by PYR and BiC) and gamma is enhanced (by PYR and PV), leading to theta-gamma coupled rhythms. In essence, the disinhibitory effect of CCK to PYR plays a controlling role in the two-phase process. Overall, we suggest that the mechanisms portrayed in Figure 6C underlie theta-gamma coupled rhythms and could serve as a motif “template” for the existence of coupled rhythms of high and low frequencies in the brain.

## DISCUSSION

### Summary and Predictions

Combining theoretical and numerical insights of a four-cell circuit model of CA1 hippocampus, we have presented a mechanism for how theta-gamma coupled rhythms arise, showcasing the contribution of different inhibitory cell types. Using a mean field model with distinct cell types, our analyses allowed us to present circuit motifs underlying the coexpression of high- and low-frequency rhythms. Gamma rhythms in theta-gamma coexpression involve a three-cell circuit motif (Figure 6). For only gamma, the theoretical work pointed to either PYR and PV or CCK and PV circuits as the gamma generators. Further, removal of PV → PV or PV → PYR connections abolished gamma rhythms, indicating support for either PING or ING type mechanisms (Whittington et al., 2000).

From our systematic numerical explorations together with theoretical analyses, we predicted that PYR and BiC underlie theta rhythm generation as they showed the largest modulation in theta power when stimulated (see Figure 4A). We had previously postulated that an “inhibition-based tuning” was responsible for the generation of theta rhythms (Skinner, Rich, Lunyov, Lefebvre, & Chatzikalymniou, 2021), and our work here predicts that BiC are the inhibitory cell type that can initiate this generation. PYR and PV showed the most modulation when theta frequency was considered (see Figure 4C). This is consistent with our previous work using detailed circuit models where we had found that inputs to PYR were able to set the population theta rhythm frequency, increasing with larger net excitatory drive (Chatzikalymniou et al., 2021). Here, with our PRM, we found that theta frequency is most strongly affected by PYR stimulation, increasing when PYR were stimulated, that is, an increased net excitatory drive.

It is known that CCK is a cell type that is highly modulated (Freund & Katona, 2007). In particular, it is known that CCK expresses highly asynchronous postsynaptic potentials (Daw, Tricoire, Erdelyi, Szabo, & McBain, 2009), making it notable that we found that the CCK → PV connection type is more variable relative to other connection types for theta-gamma coupled rhythms (see Figure 2B). From our theoretical work using two-cell subsystems, we had suggested that CCK initiated gamma rhythms, and from our numerical explorations, we found that CCK is needed to initiate theta-gamma coupled rhythms from quiescent states. Recent experimental studies have shown alternating sources of perisomatic inhibition onto pyramidal cells during behavior via CCK-expressing and PV-expressing basket cells involving inhibitory mechanisms between these cell types (Dudok et al., 2021). Theta-gamma coupled rhythms are expressed during movement exploration and REM sleep (Buzsáki, 2011), and thus, it would be interesting to examine whether these coupled rhythms are differentially affected by perturbing either PV-expressing or CCK-expressing basket cells, and whether CCK-expressing basket cells play a more robust role relative to other inhibitory cell types in the expression of theta-gamma coupled rhythms. Our results here predict that the removal of CCK-expressing basket cells should drastically reduce the presence of theta-gamma coupled rhythms, although perhaps not eradicate it, and the regularity of the theta rhythms should be reduced. Further, we predict that the modulation of CCK-expressing basket cells could lead to theta-gamma coupled rhythms being turned on or off, thus acting to control the “mood” (as borrowed from the Freund [2003] terminology) of the system.

### Related Studies

A recent data-driven modeling study probed the theta-gamma phase amplitude coupling (PAC) in hippocampal circuits focusing on three cell types—pyramidal cells, PV-expressing basket cells, and OLM cells (Ponzi, Dura-Bernal, & Migliore, 2023). Multicompartment detailed cellular models were used along with short-term synaptic plasticity, allowing the authors to highlight these cell types and particular aspects. Their results indicated a dependence of theta oscillation expression on gamma for the ubiquity of the PAC output via these cell types and their interconnections. Specifically, they showed that the theta rhythm frequency and strength relied on a PING mechanism. Our PRM is supportive of PING-like mechanisms and is thus consistent with Ponzi et al. (2023). However, due to the limited nature of our LFP representation, we did not apply PAC measures to them in the PRM.

Very detailed network models with explicitly identified and characterized cell types have been developed and can express theta and gamma rhythms (Bezaire et al., 2016; Ecker et al., 2020; Romani et al., 2024). Although it is not possible to parse mechanisms using these models, they can be used to explore biological intricacies such as ion channel complements in different cell types and receptor densities. Theta-gamma oscillations during neurostimulation has recently been explored (Vardalakis, Aussel, Rougier, & Wagner, 2024), but the theta rhythms were imposed on the hippocampal system. For experimental linkage design considerations, it may be interesting to explore neurostimulation protocols with the PRM generating theta-gamma coupled rhythms.

### Limitations and Future Work

Several biological aspects are not directly represented in the PRM. This includes aspects such as spatial inhibitory distributions and inputs onto pyramidal cells, short-term plasticity, and the inclusion of additional inhibitory cell types. The results from our study do not of course imply that these other aspects are not important. Rather, our results are limited by the cell types and interconnections that were included (Figure 1B). Further, as our model is not a spiking one, it is not able to include important spike timing considerations underlying spike-timing-dependent plasticity (STDP). A possible next step is to take advantage of Hippocampome 2.0 (Wheeler et al., 2024) to build “spiking models” that consider our PRM insights and then to be able to include STDP.

It may be possible to use our existing PRM as a starting base to consider additional cell-type contributions. For example, the contribution of OLM cells could be initially considered in an indirect way through its effect on bistratified cells by considering a decreasing STIM (inhibitory input) onto them—the PRM would indicate an enhanced theta power (see Figure 4A). Specific known motifs that include vasoactive intestinal polypeptide-expressing cell types (Guet-McCreight, Skinner, & Topolnik, 2020) can also be considered indirectly. Interneuron-specific 3 cells (VIP-expressing subtype) are known to differentially inhibit OLM cells, bistratified cells, and basket cells (Tyan et al., 2014), and so an initial consideration of inputs received by these different cell types and their effect on theta-gamma coupled rhythms could be probed. Also, as theta frequency and behavioral correlates are known to vary across the dorsoventral axis (Strange, Witter, Lein, & Moser, 2014), we can consider leveraging our models to understand these differences from particular cell types and interconnection variations. In general, one could envision expanding the PRM to directly include additional cell-type populations and connectivities and interpretations to develop further hypotheses and obtain predictions for experimental examination.

The relative strength of connections between inhibitory populations seems to change across the septotemporal axis of the CA1 hippocampus and across layers (Navas-Olive et al., 2020; Soltesz & Losonczy, 2018). Navas-Olive et al. (2020) have shown that PV-expressing basket cells preferentially innervate pyramidal cells at the deep sublayers while CCK-expressing basket cells are more likely to target the superficial pyramidal cells. Also, there is a different innervation of pyramidal cells by bistratified and OLM cells for deep versus superficial pyramidal cells. We can start to consider how these differential connectivities affect the theta-gamma coupled rhythm control by focusing on such perturbations in the PRM. Interestingly, we note that when connections from PV-expressing basket cells to pyramidal cells are larger (smaller), there can be a smaller (larger) theta frequency (see Supporting Information Table S1, last column), suggesting theta frequency differences between deep and superficial layers due to particular connection weight differences.

The role of neuromodulators such as acetylcholine in generating and modulating theta and gamma oscillations in the hippocampus has long been known (Colgin, 2013, 2016; Colgin & Moser, 2010). For example, experimental and modeling studies attempted to disentangle cell and circuit contributions to cholinergic-induced changes to oscillatory output (Tiesinga, Fellous, José, & Sejnowski, 2001). The excitability of pyramidal cells as dictated by their biophysical conductances was examined in particular. While we did not explicitly consider excitability changes in our PRM, one can envision considering this via the *β* parameter as a representation of cell excitability. As shown in Figure 3D, both theta and gamma frequencies increase with increasing *β* in the two-cell subsystems, and this could be explored in the full four-cell system.

The PRM leverages a long history of Wilson-Cowan-type models to examine the emergence of oscillatory activity in the presence of multiple interneuronal populations. Such a framework has been successfully used in the past to study a variety of different (nonhippocampal) neural circuits, enabling the mechanistic study of interactions between different excitatory and inhibitory cell types, with applications using electrophysiological and neuroimaging data (e.g., Bryson, Berkovic, Petrou, & Grayden, 2021; Hahn, Kumar, Schmidt, Knösche, & Deco, 2022; Lee, Shin, Gross, & Cho, 2018). Yet, mean field approaches possess limitations. As such, the value and scope of our predictions are contingent on parameter choices assisted by a genetic algorithm, aligning the model dynamics to various experimental constraints, notably the coexistence of gamma and theta rhythms within the correct frequency range, and other biophysical parameters (see the Model and Methods section and PRM Parameter Value Constraints in the Supporting Information). For the PRM used here, despite our experimental linkage of the different cell-type characterizations, further examination of the different parameters is warranted. For example, different and differential *β*s and delays could be considered to better capture the different cell types, and the formal inclusion of synaptic time constants could be considered, as well as differential milieu and intrinsic excitabilities. Additional theoretical analyses may be possible, but here, we were able to exploit stability and bifurcation analyses to extract our insights.

In closing, we would like to note the importance of model parameter values and interpretations in different model types relative to the experiment. For example, some parameters such as membrane time constant have the same interpretation in rate models or conductance-based models, whereas others such as synaptic weight have quite different “relationships” with experimental data for these different model types. We discuss this further in Model Parameter Interpretation in the Supporting Information. A novelty of our PRM is being explicit about the cell types used so that parameter interpretations can be considered for particular cell types along with future expansions.

## MODEL AND METHODS

### The PRM

The purpose of the PRM is the identification and analysis of key mechanisms involved in the generation and control of theta-gamma coupled rhythms, that is, coupling interactions between theta and gamma rhythms, in hippocampal microcircuits, following the formalism of population-scale mean field models. The PRM is a Wilson-Cowan-type model (H. R. Wilson & Cowan, 1972) whose dynamics are known to be dominated by (slow, 10–100 ms) synaptic inputs (Cook, Peterson, Woldman, & Terry, 2022; Deco, Jirsa, Robinson, Breakspear, & Friston, 2008). This is a consequence of the coarse time graining used in the derivation of these types of models, relying on mean field approaches and embedded assumptions (reviewed, for instance, in Chow & Karimipanah, 2020; Coombes & Wedgwood, 2023; Cowan, Neuman, & van Drongelen, 2016). Consequently, Wilson-Cowan-type models (such as the PRM) are especially relevant to describe the evolution of LFPs.

The PRM describes the time evolution of PYR, BiC, CCK, and PV activities or mean firing rates (*r*_*PYR*_, *r*_*BiC*_, *r*_*CCK*_, and *r*_*PV*_) as a function of their mutual connectivity motif (Figure 1B), synaptic weights (*w*_*n*→*m*_, for *n*, *m* = PYR, BiC, CCK, PV), membrane rate constants (*α*_*PYR*_, *α*_*BiC*_, *α*_*CCK*_, and *α*_*PV*_), synaptic and axonal delays lumped in a single term (*τ*), cell-specific intrinsic excitability (*ie*), and how these collectively contribute to the generation of a rhythmic firing rate expression. The resulting model leads to the following set of nonlinear, delayed differential equations,

$$\alpha_{PYR}^{-1}\frac{{dr}_{PYR}}{dt}\left(t\right)=-r_{PYR}\left(t\right)+r_{0,PYR}f\left[I_{PYR}^{\tau }\left(t\right)\right]$$ (1a)

$$\alpha_{BiC}^{-1}\frac{{dr}_{BiC}}{dt}\left(t\right)=-r_{BiC}\left(t\right)+r_{0,BiC}f\left[I_{BiC}^{\tau }\left(t\right)\right]$$ (1b)

$$\alpha_{CCK}^{-1}\frac{{dr}_{CCK}}{dt}\left(t\right)=-r_{CCK}\left(t\right)+r_{0,CCK}f\left[I_{CCK}^{\tau }\left(t\right)\right]$$ (1c)

$$\alpha_{PV}^{-1}\frac{{dr}_{PV}}{dt}\left(t\right)=-r_{PV}\left(t\right)+r_{0,PV}f\left[I_{PV}^{\tau }\left(t\right)\right]$$ (1d)

where *r*_0,*m*_ (here and below, *m* = *PYR*, *BiC*, *CCK*, or *PV*) represents the maximal firing rate of the neurons. Fluctuations in mean firing rates result from the nonlinear integration of presynaptic inputs *I*_*m*_. Presynaptic inputs *I*_*m*_ scale firing rates through the firing rate response function

$$f\left[I_{m}\right]=\frac{1}{1+e^{-\beta \left(iI_{m}\right)}}$$ (2)

where *I*_*m*_ = *ie*_*m*_ + *Ĩ*_*m*_. The gain beta reflects the steepness of the response, and intrinsic excitability (*ie*_*m*_) allows cell-specific threshold, rheobase, and input resistances to be taken into consideration.

The presynaptic inputs for the four cell types considered are given by

$${Ĩ}_{PYR}^{\tau }\left(t\right)=w_{PYR\to PYR}\;r_{PYR}^{\tau }+w_{BiC\to PYR}\;r_{BiC}^{\tau }+w_{PV\to PYR}\;r_{PV}^{\tau }+{\text{STIM}}_{PYR}$$ (3a)

$${Ĩ}_{BiC}^{\tau }\left(t\right)=w_{PYR\to BiC}\;r_{PYR}^{\tau }+{\text{STIM}}_{BiC}$$ (3b)

$${Ĩ}_{CCK}^{\tau }\left(t\right)=w_{CCK\to CCK}\;r_{CCK}^{\tau }+w_{PV\to CCK}\;r_{PV}^{\tau }+{\text{STIM}}_{CCK}$$ (3c)

$${Ĩ}_{PV}^{\tau }\left(t\right)=w_{CCK\to PV}\;r_{CCK}^{\tau }+w_{PV\to PV}\;r_{PV}^{\tau }+w_{PYR\to PV}\;r_{PYR}^{\tau }+{\text{STIM}}_{PV}$$ (3d)

where $r_{m}^{\tau }$ = *r*_*m*_(*t* − *τ*). A time delay of *τ* = 5 ms is included to represent physiological axon conduction delays and synaptic activation time values. The *α*_*m*_ parameter sets the time scale at which populations respond to stimuli. Cell-specific sensitivities and control are explored by including an additional input (*STIM*_*m*_).

### Choosing the Initial PRM Parameter Set

The experimental literature resources used are online databases (Bezaire et al., 2016; Wheeler et al., 2015). Details are provided in PRM Parameter Value Constraints in the Supporting Information.

We set parameter values for membrane rate constants (*α*s) and maximal firing rates (*r*_*o*_s) of the different cell types. For the numerical explorations, *β* was fixed and set to the same value for all cell types, and *τ* was set to 5 ms as a reasonable value considering physiological axon conduction delays and synaptic activation time values (Traub, Miles, & Buzsáki, 1992). We determined *ie*s for the different cell types and a set of synaptic weights. This was done by constraining the firing order of the four cell types and then using trial and error (and model intuition) to obtain synaptic weights and specific *ie* values that allowed theta-gamma coupled rhythms to be present. See Table 1 and *Set 0* in Supporting Information Table S1 for parameter values.

**Table 1.** Parameter values for PRM

**Symbol**	**Definition**	**Value**
*β*	Response function gain	20 au
*τ*	Delay	5 ms
*α*_*PYR*_	PYR rate constant	40 Hz
*α*_*BiC*_	BiC rate constant	80 Hz
*α*_*CCK*_	CCK rate constant	40 Hz
*α*_*PV*_	PV rate constant	80 Hz
*ie*_*PYR*_	PYR intrinsic excitability	0.03 au
*ie*_*BiC*_	BiC intrinsic excitability	−1.45 au
*ie*_*CCK*_	CCK intrinsic excitability	0.8 au
*ie*_*PV*_	PV intrinsic excitability	0.5 au
*r*_0,*PYR*_	PYR maximal firing rate	40 Hz
*r*_0,*BiC*_	BiC maximal firing rate	100 Hz
*r*_0,*CCK*_	CCK maximal firing rate	60 Hz
*r*_0,*PV*_	PV maximal firing rate	100 Hz

The resulting “intrinsic” activities (i.e., when all synaptic weights are zero) are shown in Supporting Information Figure S1, where the different maximal firings and differential STIM responses are consistent cellular correlates in the literature. We note that zero synaptic weights here do not prevent an interpretation that the different cell types receive inputs from other sources, just not from the other cell types included here. That is, the interpretation of a given cell’s activity when disconnected from the other cell types in the circuit (Supporting Information Figure S1) is that its activity represents its intrinsic behavior in an existing milieu that includes inputs from any other cells not directly represented in the four-cell circuit system.

This milieu without any additional stimulation (STIM = 0) means that some of the cell types (CCK and PV) are firing close to their maximal rates. This can be considered as a bit of a prediction. That is, in order to have theta-gamma coupled rhythms, the four cell types receive input (not from CCK/PV/PYR/BiC) that allow CCK and PV to fire maximally, BiC to fire minimally, and PYR to fire at intermediate levels (see Supporting Information Figure S1). We further explored this by adjusting STIM values for all of the cells (i.e., a “milieu” change) and found that this prediction aspect remained. Specifically, the minimum and maximum STIM values for which theta-gamma coupled rhythms occur are −0.04 and 1.28, respectively, considering several different parameter sets (see Supporting Information Table S1). We can say that the prediction remains since if one views (Supporting Information Figure S1), it is clear that if the curves are shifted left (by 0.04) or right (by 1.28), the differential firing aspect of the four cells would be the same as with STIM = 0.

### Simulations

Simulations of the PRM were performed using an Euler method to numerically integrate the differential equations with a time-step of 0.001 s. Each simulation was performed for 8.0 s. The initial conditions were set to zero for all cell types. The first second of the simulations was not used in the spectral analysis to avoid the transients at the start of the simulations from affecting the analysis. The output of these simulations contains the activity of the four cell types in the model. These represent the population firing rate for the corresponding cell type.

### Frequency Bands and Power Spectral Density (PSD)

We define the low-frequency *theta* band to be between 3 and 12 Hz and the high-frequency *gamma* band to be between 20 and 100 Hz. We used a Welch periodogram of the signal and separated it into the two frequency bands. The dominant frequency and its corresponding power were calculated as the frequency where the highest peak appears in each frequency band and the PSD at that frequency.

We found that filtering the signal into the low- and high-frequency bands caused anomalies due to the harmonics of the theta signal having a higher power than the gamma signal for PYR. This is due to the large difference in power between the theta and gamma bands in the PYR activity. To circumvent the issue where the harmonics of the PYR theta activity were stronger than the PYR gamma activity, we used the periodogram of the PV activity to find where the dominant frequency in the gamma band was present. This was done because the PV activity has a stronger gamma component and, as such, the harmonics of its activity in the theta band do not produce spurious results. Using the dominant frequency in the gamma band that we derived from the periodogram of the PV activity, we find the corresponding PSD for PYR at that frequency to calculate the PSD in the gamma band.

We interpret PYR as an analog of the LFP because of the much larger prevalence of PYR compared with all the other cells in the network in the actual brain tissue and since LFP recordings are mostly done in the pyramidal cell layer where PYR cell bodies are located. Using the PYR activity as a proxy for the LFP is also motivated by the fact that the PRM—a population-scale description—lacks by design the specificity to distinguish among CA1 laminar organization or cellular compartments.

The average theta and gamma powers from the 10 selected parameter sets (see Supporting Information Table S1) were used as a reference to determine whether simulation outputs had theta and gamma rhythms of a considerable enough power. Oscillations were considered “sufficient” if both the theta and gamma powers were at least 25% of this reference. Considering both theta and gamma powers in this way ensured that coexpression was reasonably present, as would be reflected in a ratio of the two powers. Performing more sophisticated analyses of cross-frequency coupling (CFC) was not deemed appropriate for these model LFP signals as the type of CFC could take many forms such as PAC, phase-phase, or amplitude-amplitude coupling, and details of the signal shape, for example, could produce many confounds (Scheffer-Teixeira & Tort, 2022).

### Genetic Algorithm

#### Starting set of parameter values.

To start exploring the parameter space, we began with the region around the set of synaptic weights that were hand-tuned and known to exhibit theta-gamma coupled rhythms, that is, the initial PRM parameter set (*Set 0*). By mutating some of the synaptic weights, we could find more sets of synaptic weights that satisfy our hypotheses and constraints.

#### Choosing which connections to mutate.

Every time we generated a new mutated set of weights, we sampled from a Poisson distribution (mean = 2) for the number of synaptic weights to mutate—this was denoted as *n*_*mutations*_. We then randomly selected *n*_*mutations*_ weights to mutate. This was done by randomly selecting two integers from (0, 4) and then selecting the weight corresponding to the connection denoted by those integers (0 = PYR, 1 = BiC, 2 = PV, 3 = CCK). If one or more of the connections we selected did not exist in our model (e.g., *BiC* → *CCK*), we resampled an integer in the same process until we got a connection that existed.

#### Mutating the weight.

For each weight that we selected to mutate, we generated a multiplicative factor in the uniform distribution from (0.1, 2). We then multiplied the existing weight by this factor to get our new weight. This ensured that the sign of the weight never changed and, as such, an excitatory weight never became an inhibitory weight or vice versa.

#### Hypothesis testing.

A given set of synaptic weights needed to satisfy the following constraints:

- Primary: At baseline (i.e., no connections removed, and no STIM applied), the system should exhibit theta and gamma oscillations such that:
  –  the power of PYR at the most prominent frequency in the theta and gamma bands are at least 60% of that in *Set 0*.
  –  the ratio of the maximal firing rate (i.e., activity) for PV and BiC is at least 0.67.
  –  the maximal firing rate of CCK is greater than 4.
- Secondary: When a certain connection type is removed, the theta oscillations break down (Chatzikalymniou et al., 2022).
  –  These connection types are PYR → PYR, CCK → PV, PV → PYR, and BiC → PYR.
  –  This is quantified by the power of the most prominent frequency in the theta band for PYR being less than 10% of that in *Set 0* at baseline.

#### Adding to valid sets of parameters.

Using our genetic algorithm, we “mutated” the synaptic weights of a constrained parameter set and checked if the resultant new set satisfied our hypotheses. If a new set does satisfy all the hypotheses, it is added to the list of the constrained sets for the next iteration of mutations. This was continued until a target number of constrained sets was found—that we set to be 200—or a maximum number of iterations was completed. We note that if we had used a different starting set of parameter values, similar distributions of synaptic weights would be obtained (not shown).

#### *k*-Means clustering and additional constraints.

The resulting parameter sets were then clustered in the *w* parameter space using a *k-means* clustering algorithm (Pedregosa et al., 2011). We found 13 distinct clusters from the 200 parameter sets. To reduce this, we decided to only analyze the clusters where at least one set of synaptic weights produced oscillations where the ratio of the activities of PV and BiC was at least 0.70—that is, increased from 0.67 used in the primary constraints. This ratio is based on relative firing rates presented in Table 6 of Bezaire et al. (2016), and the firing rate for CCK was chosen to ensure its participation in the overall system (as noted from multiple simulations). Using this additional threshold, we discarded three of the clusters that we had found. The ratios and CCK activities for these sets are given in Supporting Information Table S1.

#### Degeneracy examination.

We further examined these multiple sets. As 200 (parameter sets) is not really large enough to take advantage of visualization techniques such as *t*-distributed stochastic neighbor embedding (*t*-SNE) plots, we undertook an exploration of the clusters and their characteristics and their comparison with (random cluster) controls (details not shown here). We found that while we could not conclusively say which subspaces were more important than others, it was clear that the clusters are representative of the degeneracy existing in the space of synaptic weights, with different regions producing similar expression of theta-gamma coupled rhythms.

### Varying STIM

To explore how each cell type would respond when perturbed, STIM applied to each cell type was varied as a form of sensitivity analysis. Specifically, the STIM to each cell type was varied between −2 and 2, keeping the STIM to all other cell types fixed at zero. A positive value of STIM would mean that a cell type was being excited more than at baseline (where no STIM is applied), while a negative value of STIM would mean that the cell type is inhibited more than at baseline.

We used 121 equally spaced points in the range of values we explored. At each STIM value, the PRM was simulated, and the theta frequency, theta power, and gamma power of the PYR activity (i.e., LFP output representation) were calculated. A line of best fit was calculated for each of the theta frequency, theta power, and gamma power to quantify their change with respect to STIM to a particular cell type (see Supporting Information Figures S3 and S4). The STIM range was considered as the difference between the largest and smallest STIM values that produced sufficient oscillations. Only points that showed sufficient oscillations (see above) were used for these calculations.

### Testing for Regularity

The system was simulated for a total of 10 s, with the same time-step as previously used. For the first 5 s, STIM to all cell types was set to zero. At *t* = 5 s, STIM_*CCK*_ = −1 was applied to silence CCK. The recording was split into two sections of 4 s each: the section from *t* = 1–5 s when CCK is active and the section from *t* = 6–10 s when CCK is silent. The first 1-s interval after the start of the simulation and after STIM_*CCK*_ = −1 was applied is ignored to not count any transient activity in the analysis. For each section, the peaks in the PYR and BiC activity were recorded. The mean, median, and std of the amplitudes of the peaks and the interspike interval were recorded. This procedure was performed for all parameter sets where applicable.

### Linear Stability Analysis

For the analysis, we switch to the following notation: The mean firing rates *r*_*PYR*_, *r*_*BiC*_, *r*_*CCK*_, and *r*_*PV*_ will be denoted by *x*_1_, *x*_2_, *x*_3_, and *x*_4_, respectively. The maximal firing rates *r*_0,*PYR*_, *r*_0,*BiC*_, *r*_0,*CCK*_, and *r*_0,*PV*_ will be denoted by *r*_1_, *r*_2_, *r*_3_, and *r*_4_, respectively. To understand the mechanism of PRM, we break it into three subsystems: PYR + BiC, PYR + PV, and CCK + PV. The first considers the dynamics of PYR and BiC cells:

$$\frac{d}{dt}x_{1}\left(t\right)=\alpha_{PYR}\left(-x_{1}\left(t\right)+r_{1}f\left[I_{PYR}^{\tau }\left(t\right)\right]\right)$$ (4a)

$$\frac{d}{dt}x_{2}\left(t\right)=\alpha_{BiC}\left(-x_{2}\left(t\right)+r_{2}f\left[I_{BiC}^{\tau }\left(t\right)\right]\right)$$ (4b)

where $I_{PYR}^{\tau }$ is as in Equation 3a with *w*_*PV*→*PYR*_ = 0 and $I_{BiC}^{\tau }$ is as in Equation 3b. The second one considers the dynamics of PYR and PV:

$$\frac{d}{dt}x_{1}\left(t\right)=\alpha_{PYR}\left(-x_{1}\left(t\right)+r_{1}f\left[I_{PYR}^{\tau }\left(t\right)\right]\right)$$ (5a)

$$\frac{d}{dt}x_{4}\left(t\right)=\alpha_{PV}\left(-x_{4}\left(t\right)+r_{4}f\left[I_{PV}^{\tau }\left(t\right)\right]\right)$$ (5b)

where $I_{PYR}^{\tau }$ and $I_{PV}^{\tau }$ are defined as in Equation 3a and Equation 3d, respectively, with *w*_*BiC*→*PYR*_ = 0 and *w*_*CCK*→*PV*_ = 0. The third subsystem considers the dynamics of PV and CCK and is given by:

$$\frac{d}{dt}x_{3}\left(t\right)=\alpha_{CCK}\left(-x_{3}\left(t\right)+r_{3}f\left[I_{CCK}^{\tau }\left(t\right)\right]\right)$$ (6a)

$$\frac{d}{dt}x_{4}\left(t\right)=\alpha_{PV}\left(-x_{4}\left(t\right)+r_{4}f\left[I_{PV}^{\tau }\left(t\right)\right]\right)$$ (6b)

where $I_{CCK}^{\tau }$ and $I_{PV}^{\tau }$ are defined as in Equations 3c–3d, respectively, with *w*_*PYR*→*PV*_ = 0.

We start the analysis with the system of Equations 4a–4b. The point ($x_{1}^{*}$, $x_{2}^{*}$) is an equilibrium point of Equations 4a–4b if there is a solution to

$${ie}_{PYR}=f^{-1}\left[r_{1}^{-1}x_{1}^{*}\right]-w_{PYR\to PYR}\;x_{1}^{*}-w_{BiC\to PYR}\;x_{2}^{*}$$ (7a)

$${ie}_{BiC}=f^{-1}\left[r_{2}^{-1}x_{2}^{*}\right]-w_{PYR\to BiC}\;x_{1}^{*},$$ (7b)

where *f*^−1^(*y*) = *β*^−1^ ln(*y*/(1 − *y*)). Note that we have dropped the additional inputs *STIM*_*PYR*_ and *STIM*_*BiC*_ from Equations 7a–7b as they were initially considered to be zero.

Denote the right-hand side of Equation 4a by *ϕ*(*x*_1_, *x*_2_) and of Equation 4b by *χ*(*x*_1_, *x*_2_). Linearizing Equations 4a–4b around the equilibrium point ($x_{1}^{*}$, $x_{2}^{*}$), we find the Jacobian matrix

$$\left(\begin{array}{cc} \frac{\partial \phi }{\partial x_{1}} & \frac{\partial \phi }{\partial x_{2}}\\ \frac{\partial \chi }{\partial x_{1}} & \frac{\partial \chi }{\partial x_{2}} \end{array}\right).$$ (8)

The first-order partial derivatives of the above matrix are

$$\frac{\partial \phi }{\partial x_{1}}=-\alpha_{PYR}+\alpha_{PYR}\;\beta \;w_{PYR\to PYR}\;x_{1}^{*}\left(1-r_{1}^{-1}x_{1}^{*}\right)≔\alpha_{PYR}\left(-1+\gamma_{11}\right)$$

$$\frac{\partial \phi }{\partial x_{2}}=\alpha_{PYR}\;\beta \;w_{BiC\to PYR}\;x_{1}^{*}\left(1-r_{1}^{-1}x_{1}^{*}\right)≔\alpha_{PYR}\gamma_{12}$$

$$\frac{\partial \chi }{\partial x_{1}}=\alpha_{BiC}\;\beta \;w_{PYR\to BiC}\;x_{2}^{*}\left(1-r_{2}^{-1}x_{2}^{*}\right)≔\alpha_{BiC}\gamma_{21}$$

$$\frac{\partial \chi }{\partial x_{2}}=-\alpha_{BiC}.$$

The solution of the linearized system is of the form (*x*_1_(*t*), *x*_2_(*t*)) = (*c*_1_, *c*_2_)*e*^*λt*^, where *c*_1_, *c*_2_ are nonzero constants, *λ* are the eigenvalues, and *t* > 0. A condition on *λ* is given by the equation

$$D\left(\lambda \right)=0,$$ (9)

where

$$D\left(\lambda \right)=\text{det}\left(\begin{array}{cc} \lambda +\alpha_{PYR}-\alpha_{PYR}\gamma_{11}e^{-\lambda \tau } & -\alpha_{PYR}\gamma_{12}e^{-\lambda \tau }\\ -\alpha_{BiC}\gamma_{21}e^{-\lambda \tau } & \lambda +\alpha_{BiC} \end{array}\right).$$ (10)

From Equation 9, we have

$$\lambda^{2}+\left(\alpha_{PYR}+\alpha_{BiC}\right)\lambda +\alpha_{PYR}\alpha_{BiC}+\left(-\alpha_{PYR}\gamma_{11}\lambda -\alpha_{PYR}\alpha_{BiC}\gamma_{11}\right)e^{-\lambda \tau }-\alpha_{PYR}\alpha_{BiC}\gamma_{12}\gamma_{21}e^{-2\lambda \tau }=0.$$

To simplify the above expression, we multiply it by *e*^*λτ*^:

$$\left(\lambda^{2}+\left(\alpha_{PYR}+\alpha_{BiC}\right)\lambda +\alpha_{PYR}\alpha_{BiC}\right)e^{\lambda \tau }-\alpha_{PYR}\gamma_{11}\lambda -\alpha_{PYR}\alpha_{BiC}\gamma_{11}-\alpha_{PYR}\alpha_{BiC}\gamma_{12}\gamma_{21}e^{-\lambda \tau }=0.$$ (11)

If *λ* = *iω*, for *ω*, a nonzero real number, the bifurcation condition is defined by the simultaneous solution of the equations Re(*D*(*iω*)) = 0 and Im(*D*(*iω*)) = 0. Substituting *λ* = *iω* into Equation 11, we conclude that the real and imaginary parts are:

ReDiω=−ω2+αPYRαBiC1−γ12γ21cosωτ−αPYR+αBiCωsinωτ−αPYRαBiCγ11 (12)

and

ImDiω=−ω2+αPYRαBiC1+γ12γ21sinωτ+αPYR+αBiCωcosωτ−αPYRγ11ω. (13)

Taking Re(*D*(*iω*)) = 0 and Im(*D*(*iω*)) = 0, we find

$$\begin{array}{l} \kappa_{1}\cos\left(\omega \tau \right)+\kappa_{2}\sin\left(\omega \tau \right)+\kappa_{3}=0\\ \kappa_{4}\cos\left(\omega \tau \right)+\kappa_{5}\sin\left(\omega \tau \right)+\kappa_{6}=0 \end{array}$$ (14)

where we have further simplified the notation as follows:

$$\begin{array}{l} \kappa_{1}≔-\omega^{2}+\alpha_{PYR}\alpha_{BiC}\left(1-\gamma_{12}\gamma_{21}\right),\;\kappa_{2}≔-\left(\alpha_{PYR}+\alpha_{BiC}\right)\omega ,\;\kappa_{3}≔-\alpha_{PYR}\alpha_{BiC}\gamma_{11},\\ \kappa_{4}≔\left(\alpha_{PYR}+\alpha_{BiC}\right)\omega ,\;\kappa_{5}≔-\omega^{2}+\alpha_{PYR}\alpha_{BiC}\left(1+\gamma_{12}\gamma_{21}\right),\;\kappa_{6}≔-\alpha_{PYR}\gamma_{11}\omega . \end{array}$$ (15)

From Equation 14, we have that

$$\sin\left(\omega \tau \right)=\frac{\kappa_{3}\kappa_{4}-\kappa_{1}\kappa_{6}}{\kappa_{1}\kappa_{5}-\kappa_{2}\kappa_{4}}≔G_{1}\left(\omega \right)$$ (16a)

$$\cos\left(\omega \tau \right)=\frac{\kappa_{2}\kappa_{6}-\kappa_{3}\kappa_{5}}{\kappa_{1}\kappa_{5}-\kappa_{2}\kappa_{4}}≔G_{2}\left(\omega \right).$$ (16b)

Notice that *G*_1_ and *G*_2_ depend only on *ω*; hence, solving Equations 16a–16b with respect to *ω*, we are able to find the frequency. Indeed, the trigonometric equation sin^2^(*ωτ*) + cos^2^(*ωτ*) = 1 implies that $G_{1}^{2}$(*ω*) + $G_{2}^{2}$(*ω*) = 1. Solving the latter equation with parameter values of *Set 0*, we found a frequency in the theta band. From Equation 16a, we may also calculate the delay *τ* as follows:

$$\tau^{k}=\frac{1}{\omega }\left(\arcsin\left(G_{1}\left(\omega \right)\right)+2k\pi \right),\;k=0,1,2,\ldots \;.$$ (17)

The smallest such value is the first bifurcation point.

We repeat the same steps as above for the stability analysis of the two other subsystems (PYR + PV and CCK + PV). In Figure 3E, we plot the bifurcation points (colored triangles) for each of the three subsystems. For delays smaller than the critical ones, the subsystems are either stable or oscillations are damping. For delays larger than the critical ones, the oscillations are periodic. The curves plotted in Figure 3E are approximations of the frequencies at given delays, as computed by Equation 17.

## SUPPORTING INFORMATION

Supporting information for this article is available at https://doi.org/10.1162/netn_a_00427.

## AUTHOR CONTRIBUTIONS

Spandan Sengupta: Conceptualization; Formal analysis; Investigation; Methodology; Software; Writing – original draft; Writing – review & editing. Afroditi Talidou: Conceptualization; Formal analysis; Investigation; Software; Writing – original draft; Writing – review & editing. Jeremie Lefebvre: Conceptualization; Funding acquisition; Investigation; Project administration; Resources; Supervision; Validation; Writing – original draft; Writing – review & editing. Frances K. Skinner: Conceptualization; Funding acquisition; Project administration; Resources; Supervision; Validation; Writing – original draft; Writing – review & editing.

## FUNDING INFORMATION

Frances Skinner, Natural Sciences and Engineering Research Council of Canada (https://dx.doi.org/10.13039/501100000038), Award ID: RGPIN-2016-06182. Jeremie Lefebvre, Natural Sciences and Engineering Research Council of Canada (https://dx.doi.org/10.13039/501100000038), Award ID: RGPIN-2017-06662.

## Supplementary Material



## TECHNICAL TERMS

P/ING (pyramidal/interneuron network gamma): A terminology that has been used in the field that refers to mechanisms by which excitatory (pyramidal) and inhibitory (interneuron) cell networks interact to produce a fast (gamma) synchronized output.
Mean field model: A mathematical model of neuronal networks in which the average output of cell populations rather than individual cells is considered.
Degeneracy: Having similar output even though the underlying parameter values are different (i.e., theta-gamma coupled rhythms output with different synaptic weights).
Coupled oscillations: A state where there is activity in different frequency bands occurring simultaneously (specifically theta and gamma).
Genetic algorithm: A way in which parameter values in a mathematical model can be varied to try to fully explore the space of parameter values.
Clustering: A type of approach (e.g., *k*-means clustering) in which groups of datasets with common features can be generated.
Stability and bifurcation analysis: An approach that allows one to identify parameter values in a nonlinear mathematical model where the dynamic output changes qualitatively.
