# Supplementary material for: Cell-type-specific contributions to theta-gamma coupled rhythms in the hippocampus
Source: Netw Neurosci. 2025 Mar 3;9(1):100–24. doi: 10.1162/netn_a_00427 (PMC11949547; doi:10.1162/netn_a_00427)
Supplement: Supplementary file 1 [file netn-9-1-100-s001.pdf]

Journal: NETWORK NEUROSCIENCE

RESEARCH

Cell-type specific contributions to theta-gamma coupled rhythms in the hippocampus

Spandan Sengupta[1,*], Afroditi Talidou[1,2,*], Jeremie Lefebvre[1,2,3,], and Frances K Skinner[1,4,**]**

[1]Krembil Brain Institute, Krembil Research Institute, University Health Network, Toronto ON Canada

[2]Department of Biology, University of Ottawa, Ottawa ON Canada

[3]Department of Mathematics, University of Toronto, Toronto ON Canada

[4]Department of Physiology, University of Toronto, Toronto ON Canada

[*]These authors contributed equally to this work

[**]These senior authors contributed equally to this work

Supporting Information

- ***Supplementary Text***
- ***Six (6) Supplementary Figures***
- ***Five (5) Supplementary Tables***

SUPPLEMENTARY TEXT

PRM parameter value constraints

As noted in the Model and Methods section in the main text, we set PRM parameter values based on the experimental literature.

We used two main resources:

1. Online database – hippocampome.org (Wheeler et al., 2015), recently updated to v2.0 (Wheeler et al., 2024)
2. Table 6 and Appendix 1 of Bezaire, Raikov, Burk, Vyas, and Soltesz (2016)

We set parameter values for maximal firing rates and membrane time constants for the four cell types as follows.

From **1.**, the reported membrane time constants where available are:
representative value +/- SD (msec), [number of measurements; min, max]:

- PYR: 22.5 +/- 1.5 [20; 13.4, 39]
- PV: 13 +/- 8 [15]
- CCK: 25.07 +/- 5.6 [5]
- BiC: none reported

From **2.** (Appendix Table 1), the reported membrane time constants are (msec):
(number of measurements, animal)

- PYR: 21.5 +/- 8.6 (17, mouse)
- PV: 13.3 +/- 5.4 (7, mouse)
- CCK: 56.0 (1, rat)
- BiC: 12.2 +/- 0.6 (3, mouse)

Given this, we reasonably chose the rate constants (msec) as:

- PYR: 25
- PV: 12.5

- CCK: 25 *(focused on resource 1. as was more robust in the number of measurements)*
- BiC: 12.5

Table 1 in the main text shows the chosen membrane rate constants (inverse of time constants), α's (Hz), as [PYR, PV, CCK, BiC] = [40, 80, 40, 80].

From **1.**, the reported maximal firing rates where available are:
representative value +/- SD (Hz), [number of measurements; min, max]:

- PYR: 32 [1; 13, 200]
- PV: greater than 60 [1]
- CCK: greater than 160 [1; 28, 160]
- BiC: none reported

From **2.** Appendix, maximal firing rates (Hz) are considered from experimental recordings of firing rates plotted versus injected current for the different cell types:

- PYR: $\approx$40 (figure 5)
- PV: $\approx$100 (figure 19)
- CCK: increasing beyond 8 (figure 11). However, it should be less than PV which is 'fast-spiking' and CCK is regular-spiking (see Freund (2003)
- BiC: $\approx$100 (figure 9)

Given this, we reasonably chose the maximal firing rates (Hz) as:

- PYR: 40
- PV: 100
- CCK: 60 *(relied mainly on Freund reference as wide range reported in resource 1. and resource 2. was limited)*
- BiC: 100

Table 1 in the main text shows the chosen maximal firing rates, r_0's (Hz), as [PYR, PV, CCK, BiC] = [40, 100, 60, 100].

The other cell-specific parameter used in the model is what we term the intrinsic excitability (*ie*). We considered differences in input resistances and rheobase/threshold measurements in setting these values. A larger input resistance for cell type A relative to cell type B means that cell type A fires more easily (i.e., less leaky) relative to cell type B when depolarized. The rheobase is the least amount of depolarized current needed to result in cell spiking (as specified in Bezaire et al. (2016)'s Appendix), so it is a comparable consideration with the input resistance. No rheobase measurements are available in **1.** The threshold measurements in **1.** and **2.** were less clear for cellular differences and so we did not focus on their values.

From **1.,** the reported input resistances (Mohm) where available are:
representative value +/- SD (msec), [number of measurements; min, max]:

- PYR: 65.6 +/- 4.4 [20; 55.4, 107]
- PV: 116 +/- 63 [15]
- CCK: 281.68 +/- 79.7 [5]
- BiC: none reported

From **2.** (Appendix Table 1), the reported input resistances (Mohm) are:
(number of measurements, animal)

- PYR: 139.5 +/- 38.8 (17, mouse)
- PV: 65.2 +/- 16.2 (7, mouse)
- CCK: 298.1 (1, rat)
- BiC: 109.1 +/- 30.5 (3, mouse)

From **2.** (Appendix Table 1), the reported rheobases (pA) are:
(number of measurements, animal)

- PYR: 182.4 +/- 55.7 (17, mouse)
- PV: 307.1 +/- 109.7 (7, mouse)
- CCK: 60.0 (1, rat)
- BiC: 333.3 +/- 57.7 (3, mouse)

Given these measurements, it seems clear that CCK fires the most easily (smallest rheobase and largest input resistance values), and BiC the least, considering that it has the largest rheobase and a relatively small input resistance. For PV and PYR, the ordering would differ if one considered values from **1.** or **2.** – *we focused on resource 1. as considered more robust in the number of measurements.*

Thus, the order of (intrinsic) firing from the easiest upward, as can be seen by the relative STIM values for the activity (firing rates) in **Figure S1**, is set to be: CCK, PV, PYR, BiC. As noted in the Model and Methods section of the main text, for the *specific* values of *ie*'s, we determined them (by trial and error – 'manual tuning', as well as some model intuition) for the different cell types and a set of synaptic weights that allowed the system to produce theta-gamma coupled rhythms (initial parameter set, *Set 0*). That there is a wide difference between the rheobase of PYR and BiC can be seen as supportive of the large 'gap' between PYR and BiC firings in **Figure S1**. This firing order was maintained due to the constraint considerations described above.

Table 1 in the main text shows the chosen *ie*'s (au) as [PYR, PV, CCK, BiC] = [0.03, 0.5, 0.8, -1.45].

It is interesting to consider that the specific values we used are representative of a 'milieu' that the cells are residing in. We noted this in the Model and Methods section of the main text by stating *"We note that zero synaptic weights here do not prevent an interpretation that the different cell types receive inputs from other sources, just not from the other cell types included here. That is, the interpretation of a given cell's activity when disconnected from the other cell types in the circuit (see **Figure S1** in Supporting Information) is that its activity represents its intrinsic behaviour in an existing milieu that includes inputs from any other cells not directly represented in the four-cell circuit system."*

In the main text, the explicit *ie* values in **Table 1** were not changed in the simulations. As well, β and τ values in the simulations had set values as given in **Table 1** of the main text. Although limiting, this allowed us to extensively explore the model system both numerically and theoretically and to have clear interpretations in obtaining a mechanistic understanding of theta-gamma coupled rhythm generation. As noted in the Discussion of the main text, we think that further examination of these values should be explored, and this can be done in light of our mechanistic understandings.

PRM hypotheses and experimental constraints

(Primary constraint) Ratio of PV and BiC maximal firing rates - Table 6 in Bezaire et al. (2016) includes firing rates during theta rhythms, and it can be seen that during awake (non-anesthesized) states, BiC fires at a higher rate relative to PV. Hence, the PV/BiC ratio constraint used in the genetic algorithm was set to 0.67 (see Model and Methods in main text).

(Primary constraint) A maximal CCK firing rate of at least 4 – This value was decided on after examining many simulations and wanting to ensure that CCK could potentially contribute to circuit output. That is, if we allowed very small CCK activities to be part of the database, then one could argue that CCK was not involved at all. As shown in **Figure S2** (and described in main text), changing this maximal CCK firing rate affects the distributions, as would be expected. In the end, our uncovering of the mechanism and predicted motifs (see **Figure 6** in main text) explains why our simulations could produce such a range of CCK activity values – CCK is needed to initiate the coupled rhythms, but not necessarily required to maintain it (see main text).

(Secondary constraint) regarding loss of theta with particular connections being removed. These observations can be found in Figure 2 of Chatzikalymniou, Sengupta, Lefebvre, and Skinner (2022) that uses simulations of the full-scale model (FSM) of Bezaire et al. (2016) in which connections are removed by setting the particular synaptic conductances in the FSM to zero. In the original paper (Bezaire et al., 2016), simulations in which particular cell types were removed was done, but not simulations in which particular connections were removed. In Chatzikalymniou et al. (2022), connection removal was carried out to try to identify which connection(s) were essential for the presence of theta oscillations. We used those results to constrain PRM parameter values in its ability to express theta rhythms – an inability to express theta would in turn prevent theta-gamma coupled rhythms from being expressed.

Model parameter interpretation

The over-arching goal is to understand the biological system and its dynamics in different contexts. Help toward this goal requires mathematical modeling, but many different model types are used. As such, clear interpretation of parameters relative to the biological system is essential.

Interpretation of model parameters with experimental data and their corresponding values in different model types (i.e., rate models, spiking models, conductance-based models, multi-compartment models etc.) varies. Some parameter interpretations are clear and are the same across different model types (e.g., membrane time constant). However, others such as synaptic weight are fully dependent on the cell model and connection design specifics. If one is using a detailed multi-compartment model, then 'synaptic weight' can be interpreted in a straightforward fashion relative to experiment in terms of consideration of density of synapses, location, conductance, and so on, depending on the level of detail that is included in the multi-compartment model. For a rate model, such as PRM used here, the synaptic weight is not easily interpreted relative to experimental values. For example, a larger synaptic weight in PRM does not necessarily directly translate to a larger synaptic conductance value in the biological system, as the synapses could be distally or proximally located (as one potential factor) which would affect their effective 'synaptic weight' – many biological aspects are lumped in the synaptic weight parameter and the 'lumping' depends on biological cell details that are not part of these models. While one may not be able to directly compare synaptic weights with experiment, one can take insight from the model of relative synaptic weights and contribution to circuit output, for example. The advantage of model types like PRM is that mathematical analyses become possible as well as being able to do a more thorough parameter space exploration.

In this work, we 'linked' the detailed Bezaire model with PRM in the choice of cell types and their connections along with 'mechanistically' linking the requirement that theta oscillations are lost when certain connections are removed (i.e., secondary constraint). As the Bezaire model is directly linked with many experimental values, the assumption in this linkage is that the Bezaire model behaviour is reasonably representative of the biological system in this way. However, we consider this as a one-way link. That is, once we created our PRM with its cell types, connections and obtained parameter values constrained as described, we could fully analyze PRM and obtain insights and predictions regarding theta-gamma coupled rhythms with its given cell types and connections. At that point, we would ***not*** go back to the Bezaire model to explore PRM predictions, but rather, we would consider the biological system itself to directly assess our predicted model mechanisms and insights where possible. A novelty of our PRM is being explicit about the cell types used so that parameter interpretations can be considered for particular cell types. Moving forward, one could consider expanding PRM to include additional cell

types and/or additional connections (see Discussion in main text). One could also consider creating spiking models based on PRM insights.

REFERENCES

Bezaire, M. J., Raikov, I., Burk, K., Vyas, D., & Soltesz, I. (2016). Interneuronal mechanisms of hippocampal theta oscillation in a full-scale model of the rodent CA1 circuit. *eLife*, *5*, e18566. doi: 10.7554/eLife.18566

Chatzikalymniou, A. P., Sengupta, S., Lefebvre, J., & Skinner, F. K. (2022). Cholecystokinin-expressing (CCK+) basket cells are key controllers of theta-gamma coupled rhythms in the hippocampus. *bioRxiv*. (Pages: 2022.06.01.494440 Section: New Results) doi: 10.1101/2022.06.01.494440

Freund, T. F. (2003). Interneuron Diversity series: Rhythm and mood in perisomatic inhibition. *Trends in Neurosciences*, *26*(9), 489–495. doi: 10.1016/S0166-2236(03)00227-3

Wheeler, D. W., Kopsick, J. D., Sutton, N., Tecuatl, C., Komendantov, A. O., Nadella, K., & Ascoli, G. A. (2024). Hippocampome.org 2.0 is a knowledge base enabling data-driven spiking neural network simulations of rodent hippocampal circuits. *eLife*, *12*. doi: 10.7554/eLife.90597

Wheeler, D. W., White, C. M., Rees, C. L., Komendantov, A. O., Hamilton, D. J., & Ascoli, G. A. (2015). Hippocampome.org: a knowledge base of neuron types in the rodent hippocampus. *eLife*, *4*, e09960. doi: 10.7554/eLife.09960

SUPPLEMENTARY FIGURES

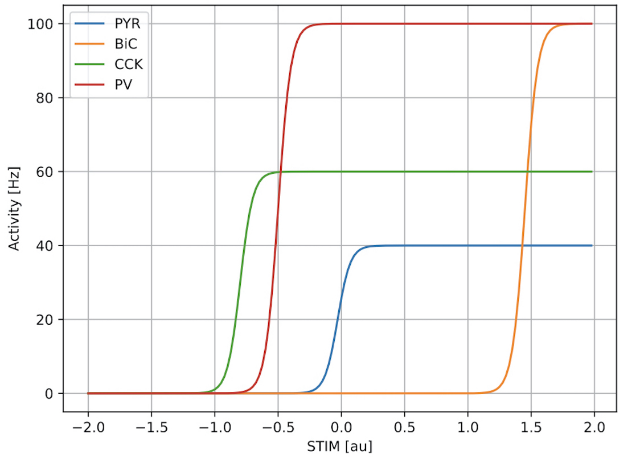

190 **Figure S 1. Intrinsic firing rates for the four cell types in the PRM.**

191 As STIM is varied, the firing of the four cell types changes as shown.

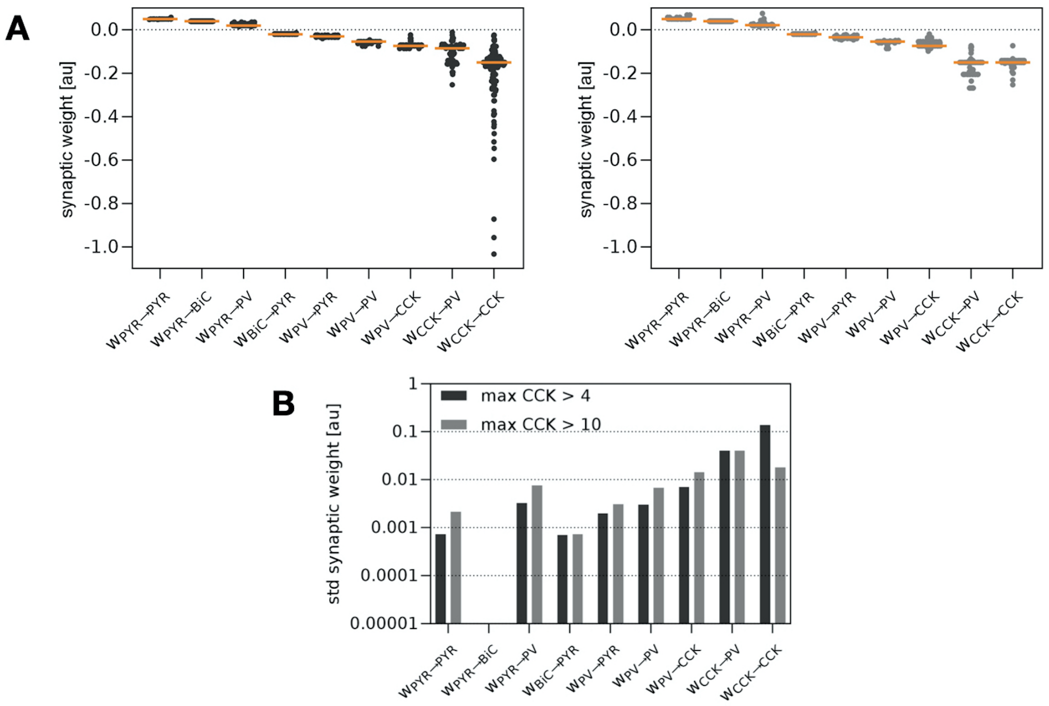

Figure S 2. Additional distributions.

A. Distribution of synaptic weights (w) for each of the nine different connections in the PRM, from 200 constrained parameter sets of w found when running the genetic algorithm for a maximal firing of CCK constrained to be greater than 4 (**left**) or greater than 10 (**right**). The orange line depicts the median.

B. Bar graph showing the standard deviations (std) of w for each of the nine different connections when maximal firing of CCK was either 4 or 10 as labelled. As expected, maximal CCK firing greater than 4 or 10 results in different w's, but the relative sizes of w remained the same, and the CCK→PV connection remained as a w with one of the largest std.

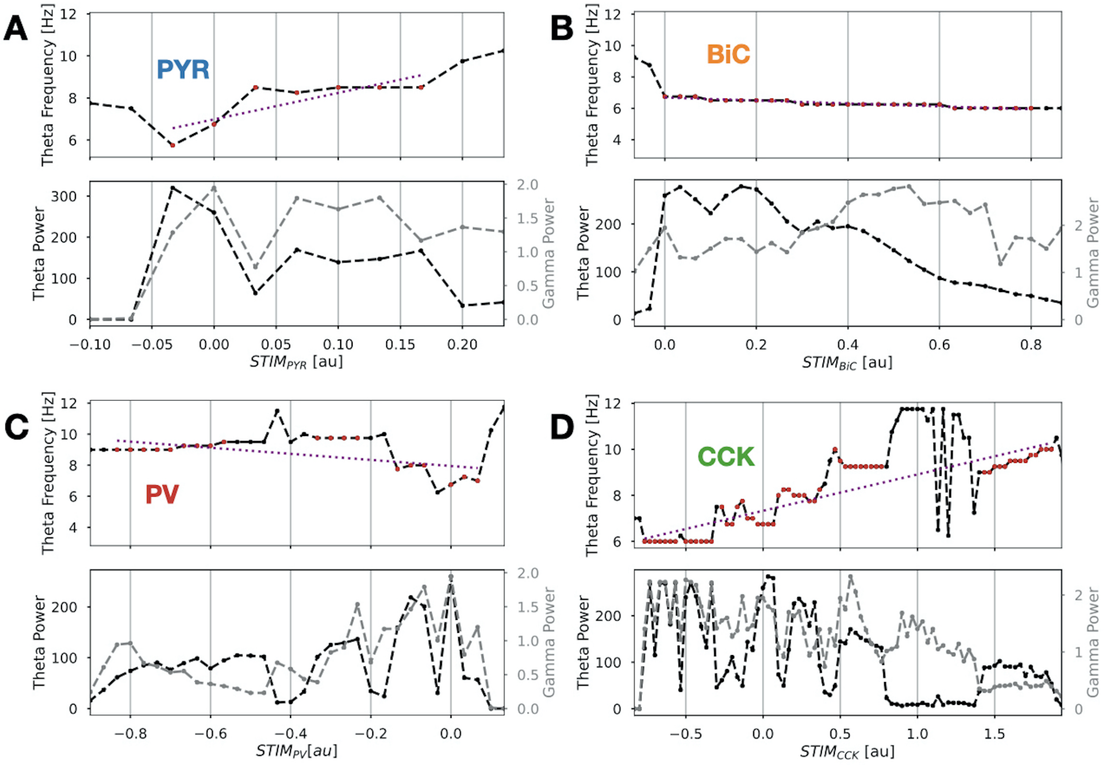

Figure S 3. Example illustration of slope quantification for theta and gamma powers and theta frequency with STIM variation.

LFP theta frequency (top parts) and theta and gamma power (bottom parts) in the four-cell system with varying STIM to PYR (**A**), BiC (**B**), PV (**C**), and CCK (**D**) cell types are plotted using *Set 4* parameter values. The plots are truncated to be centred around the values of STIM that allow the system to exhibit theta-gamma coupled rhythms, and one can immediately see that there is a wider range (i.e., difference between the maximum and minimum) of STIM values for CCK relative to the other cell types. This is summarized in *Figure 4**D*** in main text. The STIM values where sufficient theta-gamma coupled rhythms occur are marked using red dots on the theta frequency plots. The dashed purple line shows the line best fitting the variation of theta frequency with STIM using only the points (red dots) where theta-gamma oscillations occur. Similar lines (not shown) and fits were done for theta and gamma powers. The slopes of the theta and gamma power line fits are used to make the plots shown in *Figure 4**A, B*** in main text. The plot using the slopes of the theta frequency line fits is shown in *Figure 4**C*** in main text. We note that changes in theta and gamma powers and theta frequency do not necessarily change in a smooth fashion. This jaggedness is due to the dynamic, nonlinear coupling interactions occurring between theta and gamma rhythms which affects the amplitude and expression of the theta-gamma coupled rhythms as STIM is varied, and hence theta and gamma powers and theta frequency. We illustrate this in *Figure S4*.

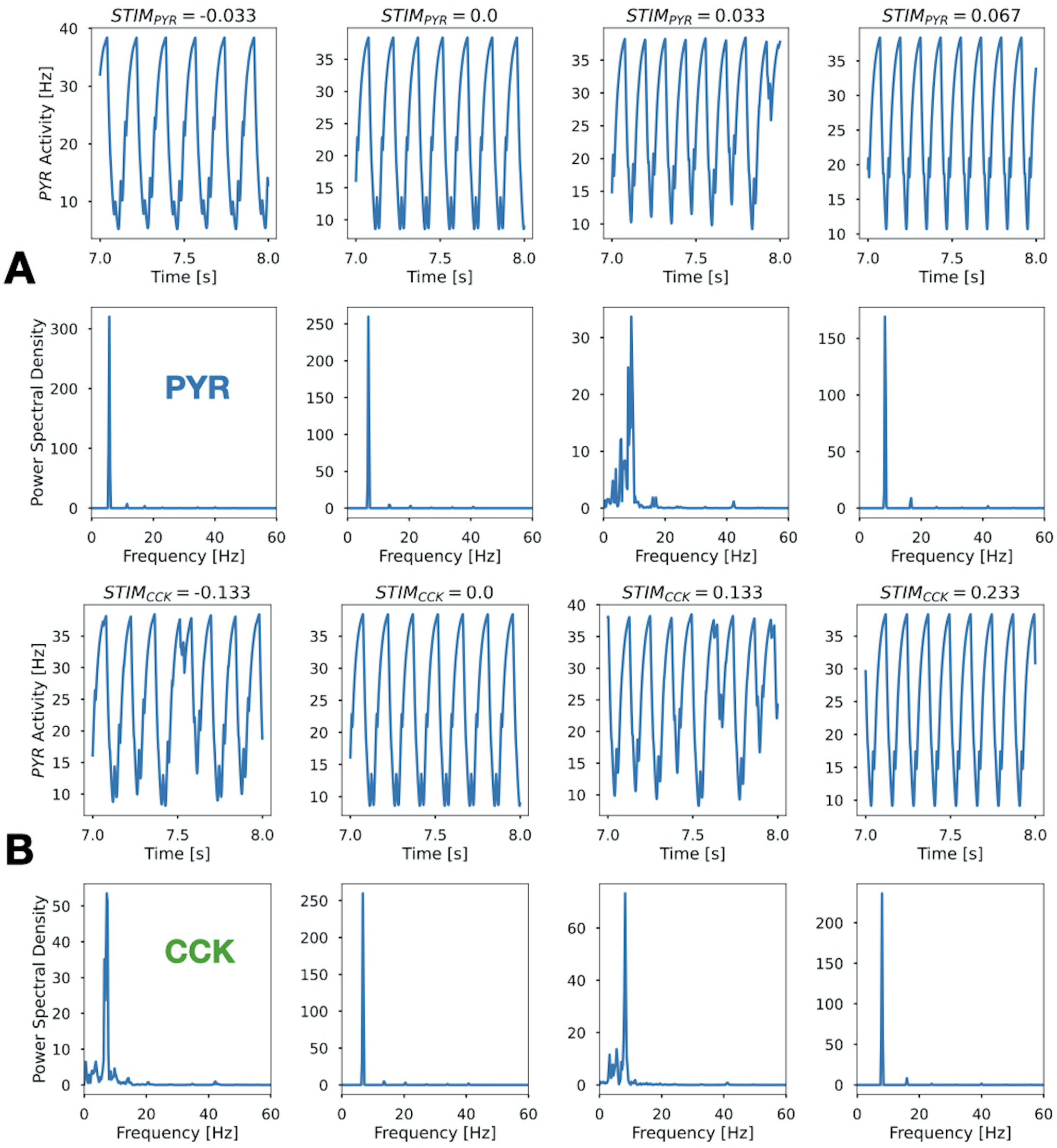

Figure S 4. Example illustration of PYR activities and their power spectral densities.

PYR activity outputs for four different STIM values and the power spectral density (PSD) associated with the PYR activity (LFP representation). **A.** STIM values when applied to PYR **B.** STIM values when applied to CCK. This is for *Set 4* parameter values, representing specific red dots shown in *Figure S3*. From these selected examples, it is clear why there is jaggedness in the theta and gamma powers and theta frequency. In viewing the PSDs, it is clear that the theta powers are much larger than the gamma powers. PSD units are Hz.

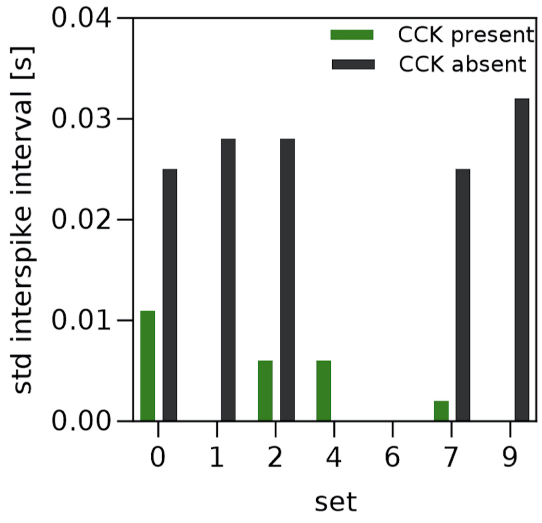

214 **Figure S 5. Changes in regularity of theta events.**

215 Bar graph showing how the theta rhythm regularity is affected by the presence of absence of CCK during ongoing theta-gamma coupled rhythms for seven of 216 the ten sets as labelled. If no bar is present, then the standard deviation (std) is zero. The specific values are given in *Table S3*.

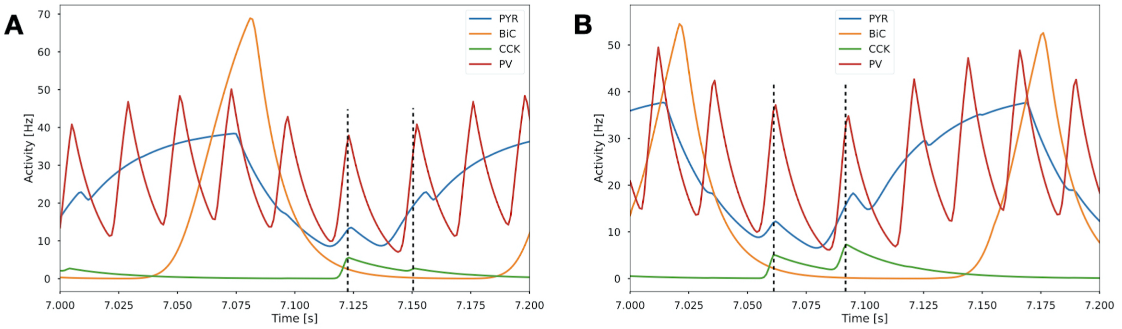

217 **Figure S 6. A blow up of activities to illustrate timings.**

218 Activities of the four cell types are shown for a 0.1 second range for *Set 4* parameter values **(A)**, and *Set 5* parameter values **(B)**. Vertical lines show that the 219 peaks of CCK precede both PV and PYR, thus illustrating support for the two-phase process schematized in *Figure 6**C*** (in main text) where CCK initiates the 220 gamma in the first phase.

SUPPLEMENTARY TABLES

221 **Table S 1. Synaptic weight values and characteristics for the ten selected parameter sets**

**Set**	$w_{PYR \to PYR/BiC/PV}$ *(au)*	$w_{BiC \to PYR}$ *(au)*	$w_{CCK \to CCK/PV}$ *(au)*	$w_{PV \to PV/PYR/CCK}$ *(au)*	*PV/BiC ratio*	*max CCK (Hz)*	*Theta freq (Hz)*
0	0.05/0.04/0.02	-0.02	-0.15/-0.15	-0.055/-0.03/-0.075	0.71	10.11	7.75
1	0.05/0.04/0.02	-0.02	-0.15/-0.107	-0.055/-0.03/-0.075	0.71	6.29	6.5
2	0.05/0.04/0.02	-0.019	-0.15/-0.15	-0.055/-0.03/-0.075	0.71	8.08	8.0
3	0.05/0.04/0.036	-0.02	-0.15/-0.107	-0.055/-0.03/-0.075	1.05	7.38	4.5
4	0.05/0.04/0.02	-0.019	-0.15/-0.078	-0.055/-0.03/-0.075	0.72	5.71	6.75
5	0.05/0.04/0.02	-0.02	-0.15/-0.11	-0.055/-0.035/-0.075	0.83	8.18	6.5
6	0.05/0.04/0.02	-0.02	-0.15/-0.024	-0.055/-0.025/-0.075	0.71	4.76	8.5
7	0.05/0.04/0.02	-0.02	-0.48/-0.078	-0.055/-0.03/-0.075	0.73	5.45	6.75
8	0.05/0.04/0.027	-0.019	-0.27/-0.078	-0.055/-0.03/-0.052	0.85	9.92	7.0
9	0.05/0.04/0.02	-0.019	-1.03/-0.086	-0.055/-0.03/-0.071	0.72	5.46	6.75

All of the synaptic weight values for the ten parameter sets are given. Other parameter values are given in ***Table 1*** *(in main text). Unless otherwise indicated, STIM is set to zero for all cell types. Also shown are the activity ratios of PV/BiC and the maximal CCK activities which formed part of the constraints (see details in Model and Methods section), as well as the theta frequency, for each of the ten parameter sets.*

222 **Table S 2. Theta and gamma power changes with connection removals**

**Set**	*Connection type removal*	*Theta power ratio*[1]	*Gamma power ratio*[1]	*Sufficient?*[2] *(Y/N)*
0-3,5	PYR→PYR*	≈ 0	> 0.2	N
4	PYR→PYR*	≈ 0	0.06	N
6-9	PYR→PYR*	≈ 0	< 0.05 (≠ 0)	N
0-9	PYR→BiC	≈ 0	≈ 0	N
0,2	PYR→PV	≈ 0	≈ 0	N
1,3	PYR→PV	> 0.2	> 0.2	N
4-9	PYR→PV	> 0.2	> 0.2	**Y**
0-9	BiC→PYR*	≈ 0	≈ 0	N
0-9	PV→PYR*	≈ 0	≈ 0	N
0-9	PV→PV	≈ 0	≈ 0	N
0,2,3,5	PV→CCK	< 0.07 (≠ 0)	> 0.2	N
1	PV→CCK	0.11	> 0.2	N
4	PV→CCK	0.17	> 0.2	N
6,9	PV→CCK	> 0.2	> 0.2	**Y**
7	PV→CCK	> 0.2	> 0.2	N
8	PV→CCK	0.12	> 0.2	N
0-9	CCK→PV*	≈ 0	< 0.01 (≠ 0)	N
0-5,7-9	CCK→CCK	≈ 0	≈ 0	N
6	CCK→CCK	> 0.2	> 0.2	**Y**

[1] *Theta or gamma power ratio is the ratio of the theta power when the particular connection is removed divided by the reference theta or gamma power when no connections are removed.*

[2] *Sufficient means that both theta and gamma powers are large enough to be considered to have theta-gamma coupled rhythms to be present. $>25\%$ of the reference power was used as the threshold. Note that if $>20\%$ was used, more cases would have been considered to be sufficient.*

Due to applied constraints (see* ***Figure 1 E-H* *in main text), removal of these connections is expected to yield no theta rhythms, and hence no theta-gamma coupled rhythms. See Model and Methods section for further details.*

223 **Table S 3. Changes in amplitude and interspike intervals of LFP theta with and without CCK**

**Set**	*Amplitude (au)*		*Mean ISI (s)*		*Std ISI (s)*	
	CCK present	*CCK absent*	*CCK present*	*CCK absent*	*CCK present*	*CCK absent*
0	38.2	38.1	0.128	0.147	0.011	0.025
1	38.5	37.9	0.153	0.135	0.0	0.028
2	38.2	38.1	0.126	0.146	0.006	0.028
4	38.4	38.4	0.146	0.167	0.006	0.0
6	38.3	38.3	0.117	0.117	0.0	0.0
7	38.2	37.9	0.147	0.140	0.002	0.025
9	38.4	38.3	0.147	0.167	0.0	0.032

ISI = interspike interval between PYR peaks (i.e., theta cycles of LFP representation); Std = standard deviation.

224 **Table S 4. Statistical comparisons between cell types for theta and gamma power and ranges**

Theta power			***Gamma power***			**STIM range**		
Cell 1	*Cell 2*	*p-value*	*Cell 1*	*Cell 2*	*p-value*	*Cell 1*	*Cell 2*	*p-value*
PYR	BiC	6.3×10^{-2} *	PYR	BiC	1.9×10^{-2}	CCK	PYR	1.1×10^{-5}
PYR	CCK	1.1×10^{-5}	PYR	CCK	1.5×10^{-3}	CCK	BiC	2.2×10^{-5}
PYR	PV	1.1×10^{-5}	PYR	PV	4.9×10^{-4}	CCK	PV	2.2×10^{-5}
BiC	PV	1.1×10^{-5}	PV	BiC	1.9×10^{-1} *			
BiC	CCK	1.1×10^{-5}	PV	CCK	1.9×10^{-2}			
PV	CCK	1.1×10^{-5}	BiC	CCK	6.8×10^{-1} *			

** Not statistically significant. These comparisons are related to* ***Figure 4A,B,D*** *in main text. Comparisons were done using Mann-Whitney (95%, $p<0.05$).*

Table S 5. Statistical comparisons between cell types for theta frequency

	Theta frequency	
Cell 1	*Cell 2*	*p-value*
PYR	BiC	3.3×10^{-4} *
PYR	CCK	2.1×10^{-3}
PYR	PV	1.1×10^{-5}
BiC	CCK	7.5×10^{-2} *
BiC	PV	3.9×10^{-3}
CCK	PV	1.1×10^{-5}

** Not statistically significant. These comparisons are related to* ***Figure 4C*** *in main text. Comparisons were done using Mann-Whitney (95%, p<0.05).*
